# Genetic parameters and genome-wide association studies including the X chromosome for various reproduction and semen quality traits in Nellore cattle

**DOI:** 10.1186/s12864-024-11193-2

**Published:** 2025-01-10

**Authors:** Felipe E. de Carvalho, José Bento S. Ferraz, Victor B. Pedrosa, Elisangela C. Matos, Joanir P. Eler, Marcio R. Silva, José D. Guimarães, Fernando Bussiman, Barbara C. A. Silva, Henrique A. Mulim, Artur Oliveira Rocha, Andre C. Araujo, Hui Wen, Gabriel S. Campos, Luiz F. Brito

**Affiliations:** 1 Department of Veterinary Medicine, Faculty of Animal Science and Food Engineering, University of São Paulo, Pirassununga, SP Brazil; 2 Department of Animal Sciences, Purdue University, 270 S. Russell Street, West Lafayette, IN 47907 USA; 3 Department of Veterinary Medicine, Federal University of Vicosa, Vicosa, MG Brazil; 4 Department of Animal and Dairy Science, University of Georgia, Athens, GA USA

**Keywords:** GWAS, Heritability, Reproductive performance, Zebu cattle

## Abstract

**Background:**

The profitability of the beef industry is directly influenced by the fertility rate and reproductive performance of both males and females, which can be improved through selective breeding. When performing genomic analyses, genetic markers located on the X chromosome have been commonly ignored despite the X chromosome being one of the largest chromosomes in the cattle genome. Therefore, the primary objectives of this study were to: (1) estimate variance components and genetic parameters for eighteen male and five female fertility and reproductive traits in Nellore cattle including X chromosome markers in the analyses; and (2) perform genome-wide association studies and functional genomic analyses to better understand the genetic background of male and female fertility and reproductive performance traits in Nellore cattle.

**Results:**

The percentage of the total direct heritability (h^2^_total_) explained by the X chromosome markers (h^2^_x_) ranged from 3 to 32% (average: 16.4%) and from 9 to 67% (average: 25.61%) for female reproductive performance and male fertility traits, respectively. Among the traits related to breeding soundness evaluation, the overall bull and semen evaluation and semen quality traits accounted for the highest proportion of h^2^_x_ relative to h^2^_total_ with an average of 39.5% and 38.75%, respectively. The total number of significant genomic markers per trait ranged from 7 (seminal vesicle width) to 43 (total major defects). The number of significant markers located on the X chromosome ranged from zero to five. A total of 683, 252, 694, 382, 61, and 77 genes overlapped with the genomic regions identified for traits related to female reproductive performance, semen quality, semen morphology, semen defects, overall bulls’ fertility evaluation, and overall semen evaluation traits, respectively. The key candidate genes located on the X chromosome are *PRR32*, *STK26*, *TMSB4X*, *TLR7*, *PRPS2*, *SMS*, *SMARCA1*, *UTP14A*, and *BCORL1*. The main gene ontology terms identified are “Oocyte Meiosis”, “Progesterone Mediated Oocyte Maturation”, “Thermogenesis”, “Sperm Flagellum”, and “Innate Immune Response”.

**Conclusions:**

Our findings indicate the key role of genes located on the X chromosome on the phenotypic variability of male and female reproduction and fertility traits in Nellore cattle. Breeding programs aiming to improve these traits should consider adding the information from X chromosome markers in their genomic analyses.

**Supplementary Information:**

The online version contains supplementary material available at 10.1186/s12864-024-11193-2.

## Background

The demand for animal protein is expected to increase up to 21% by 2050 driven by the worldwide population growth [1]. Beef cattle is one of the main sources of animal protein in many parts of the world. Therefore, increasing productivity and production efficiency are key strategic goals to meet this growing demand for animal protein [2]. Brazilian Nellore cattle (*Bos taurus indicus*) are well adapted to harsh environmental conditions and can produce high quality protein in pasture-based systems at a relatively low cost [3]. However, the fertility and reproductive performance of Nellore animals could be substantially improved to increase the breed competitiveness, especially when compared to taurine breeds.

Genomic selection can be used for improving fertility and reproductive efficiency of Nellore cattle as these traits are heritable [4, 5]. Improved fertility and female reproductive performance will directly impact the number of offspring produced per year, lowering rates of reproductive failure, and reducing the number of culled females [6]. Previous studies have been conducted to understand the genetic background of semen characteristics [7], female fertility [8], and reproductive performance and efficiency in males and females [5]. These studies considered all available genetic and genomic information, showing significant progress in the understanding of the genetic background of reproductive performance traits in males and females, including studies also fitting genomic markers located on the X chromosome [9, 10].

When discarding the X chromosome markers in genomic analyses, one could be neglecting the effects of important X-chromosome genes on phenotypic variability for various complex traits [11]. Adding the X chromosome markers to the genomic prediction of breeding values, especially for fertility and reproductive traits, could increase the accuracy of genomic breeding values [9, 12], and consequently, the rate of genetic progress for these traits. Genes located on the X chromosome have been reported to influence reproductive functions, such as embryonic development, production of gametes, testicular development, spermatogenesis, and conception rate [13–15].

Despite its known importance on the genetic determination of fertility and reproduction traits, considering markers located on the X chromosome in genomic analyses in cattle has been challenging due to the mode of inheritance of sex chromosomes in mammals, including the fact that males are hemizygous (XY) in the non-pseudo autosomal regions, and one X chromosome (XX) is inactivated in females during early development to ensure equivalent X-linked gene expression in cells of animals from both sexes (i.e., dosage compensation effect) [9, 12, 16, 17]. Therefore, understanding the genetic contributions of genes located on the X chromosome on the phenotypic variability of fertility and reproduction traits could be paramount for optimizing beef cattle breeding programs. Therefore, the primary objectives of this study were to: (1) estimate variance components and genetic parameters for various male and female fertility and reproduction traits including X chromosome markers in the analyses; and, (2) perform genome-wide association studies and functional genomic analyses to better understand the genetic background of male and female fertility and reproduction traits in Nellore cattle.

## Results

### Heritability estimates

To our best knowledge, this is the first study including data from the X chromosome for estimating variance components and heritability for fertility and reproductive performance traits in both male and female Nellore animals. Tables 1 and 2 show the heritability estimates and variance components captured by autosomal and X chromosome loci. All standard error (SE) estimates were below 0.001.

**Table 1:** Variance components and heritability estimates, for autosomes and X chromosome estimates, highest posterior density range (HPD; 5 − 95% interval), and Geweke score for female reproductive performance traits in Nellore cattle

Traits		Mean	HPD Interval (5%, 95%)		Geweke (Z-score)
	Reproductive female traits				
**REBA**	$\:{\mathbf{h}}_{\mathbf{c}\mathbf{h}\mathbf{r}\mathbf{x}}^{2}$	**0.08**	0.04	0.11	0.15
	$\:{{\upsigma\:}}_{{\mathbf{u}}_{\mathbf{c}\mathbf{h}\mathbf{r}\mathbf{x}}}^{2}$	0.10	0.06	0.15	0.10
	$\:{\mathbf{h}}_{\mathbf{a}\mathbf{u}\mathbf{t}\mathbf{o}}^{2}$	**0.17**	0.07	0.27	0.07
	$\:{{{\upsigma\:}}_{\mathbf{u}}^{2}}_{\mathbf{a}\mathbf{u}\mathbf{t}\mathbf{o}}$	0.23	0.06	0.40	0.02
	$\:{\mathbf{h}}_{\mathbf{t}\mathbf{o}\mathbf{t}\mathbf{a}\mathbf{l}}^{2}$	0.25	0.10	0.29	0.08
**REBB**	$\:{\mathbf{h}}_{\mathbf{c}\mathbf{h}\mathbf{r}\mathbf{x}}^{2}$	**0.05**	0.01	0.10	0.64
	$\:{{\upsigma\:}}_{{\mathbf{u}}_{\mathbf{c}\mathbf{h}\mathbf{r}\mathbf{x}}}^{2}$	0.08	0.01	0.14	0.64
	$\:{\mathbf{h}}_{\mathbf{a}\mathbf{u}\mathbf{t}\mathbf{o}}^{2}$	**0.26**	0.21	0.31	0.38
	$\:{{{\upsigma\:}}_{\mathbf{u}}^{2}}_{\mathbf{a}\mathbf{u}\mathbf{t}\mathbf{o}}$	0.37	0.29	0.46	0.27
	$\:{\mathbf{h}}_{\mathbf{t}\mathbf{o}\mathbf{t}\mathbf{a}\mathbf{l}}^{2}$	**0.31**	0.22	0.32	0.64
**REB**	$\:{\mathbf{h}}_{\mathbf{c}\mathbf{h}\mathbf{r}\mathbf{x}}^{2}$	**0.05**	0.03	0.07	0.42
	$\:{{\upsigma\:}}_{{\mathbf{u}}_{\mathbf{c}\mathbf{h}\mathbf{r}\mathbf{x}}}^{2}$	0.07	0.03	0.10	0.41
	$\:{\mathbf{h}}_{\mathbf{a}\mathbf{u}\mathbf{t}\mathbf{o}}^{2}$	**0.22**	0.18	0.26	0.19
	$\:{{{\upsigma\:}}_{\mathbf{u}}^{2}}_{\mathbf{a}\mathbf{u}\mathbf{t}\mathbf{o}}$	0.30	0.24	0.36	0.15
	$\:{\mathbf{h}}_{\mathbf{t}\mathbf{o}\mathbf{t}\mathbf{a}\mathbf{l}}^{2}$	**0.27**	0.18	0.28	0.14
**PP14**	$\:{\mathbf{h}}_{\mathbf{c}\mathbf{h}\mathbf{r}\mathbf{x}}^{2}$	**0.06**	0.02	0.10	0.06
	$\:{{\upsigma\:}}_{{\mathbf{u}}_{\mathbf{c}\mathbf{h}\mathbf{r}\mathbf{x}}}^{2}$	0.14	0.04	0.23	0.05
	$\:{\mathbf{h}}_{\mathbf{a}\mathbf{u}\mathbf{t}\mathbf{o}}^{2}$	**0.42**	0.35	0.48	0.00
	$\:{{{\upsigma\:}}_{\mathbf{u}}^{2}}_{\mathbf{a}\mathbf{u}\mathbf{t}\mathbf{o}}$	1.01	0.76	1.25	-0.02
	$\:{\mathbf{h}}_{\mathbf{t}\mathbf{o}\mathbf{t}\mathbf{a}\mathbf{l}}^{2}$	0.48	0.33	0.49	-0.03
**STAY**	$\:{\mathbf{h}}_{\mathbf{c}\mathbf{h}\mathbf{r}\mathbf{x}}^{2}$	**0.01**	0.01	0.02	0.44
	$\:{{\upsigma\:}}_{{\mathbf{u}}_{\mathbf{c}\mathbf{h}\mathbf{r}\mathbf{x}}}^{2}$	0.02	0.01	0.03	0.44
	$\:{\mathbf{h}}_{\mathbf{a}\mathbf{u}\mathbf{t}\mathbf{o}}^{2}$	**0.33**	0.30	0.35	-0.02
	$\:{{{\upsigma\:}}_{\mathbf{u}}^{2}}_{\mathbf{a}\mathbf{u}\mathbf{t}\mathbf{o}}$	0.50	0.45	0.55	-0.06
	$\:{\mathbf{h}}_{\mathbf{t}\mathbf{o}\mathbf{t}\mathbf{a}\mathbf{l}}^{2}$	**0.34**	0.32	0.38	-0.05

REB: All records of rebreeding of females; REBB: Rebreeding of females that entered reproduction at two years old; REBA: Rebreeding of precocity heifers; PP14: Pregnancy probability at 14 months; STAY: Ability to remain productive in the herd. The SE for all heritability estimated was < 0.001

**Table 2:** Variance components and heritability estimates, highest posterior density interval (HPD), and Geweke score for andrological traits in Nellore cattle using separated genetic random effects for the autosome and X chromosome in the statistical models

Traits		Mean	HPD Interval		Geweke (Z-score)
			(5%	95%)	
	Bull’s Traits				
**VOL**	$\:{\mathbf{h}}_{\mathbf{c}\mathbf{h}\mathbf{r}\mathbf{x}}^{2}$	**0.02**	0.00	0.04	0.57
	$\:{{\upsigma\:}}_{{\mathbf{u}}_{\mathbf{c}\mathbf{h}\mathbf{r}\mathbf{x}}}^{2}$	0.07	0.00	0.14	0.57
	$\:{\mathbf{h}}_{\mathbf{a}\mathbf{u}\mathbf{t}\mathbf{o}}^{2}$	**0.03**	0.01	0.06	0.39
	$\:{{{\upsigma\:}}_{\mathbf{u}}^{2}}_{\mathbf{a}\mathbf{u}\mathbf{t}\mathbf{o}}$	0.11	0.03	0.19	0.39
	$\:{\mathbf{h}}_{\mathbf{t}\mathbf{o}\mathbf{t}\mathbf{a}\mathbf{l}}^{2}$	**0.06**	0.01	0.07	0.31
**MOT**	$\:{\mathbf{h}}_{\mathbf{c}\mathbf{h}\mathbf{r}\mathbf{x}}^{2}$	**0.01**	0.00	0.03	0.53
	$\:{{\upsigma\:}}_{{\mathbf{u}}_{\mathbf{c}\mathbf{h}\mathbf{r}\mathbf{x}}}^{2}$	1.65	0.26	3.55	0.53
	$\:{\mathbf{h}}_{\mathbf{a}\mathbf{u}\mathbf{t}\mathbf{o}}^{2}$	**0.03**	0.01	0.06	0.32
	$\:{{{\upsigma\:}}_{\mathbf{u}}^{2}}_{\mathbf{a}\mathbf{u}\mathbf{t}\mathbf{o}}$	4.23	1.48	6.98	0.31
	$\:{\mathbf{h}}_{\mathbf{t}\mathbf{o}\mathbf{t}\mathbf{a}\mathbf{l}}^{2}$	**0.05**	0.02	0.06	0.32
**VIG**	$\:{\mathbf{h}}_{\mathbf{c}\mathbf{h}\mathbf{r}\mathbf{x}}^{2}$	**0.02**	0.00	0.03	0.28
	$\:{{\upsigma\:}}_{{\mathbf{u}}_{\mathbf{c}\mathbf{h}\mathbf{r}\mathbf{x}}}^{2}$	0.00	0.00	0.01	0.28
	$\:{\mathbf{h}}_{\mathbf{a}\mathbf{u}\mathbf{t}\mathbf{o}}^{2}$	**0.02**	0.01	0.03	-0.15
	$\:{{{\upsigma\:}}_{\mathbf{u}}^{2}}_{\mathbf{a}\mathbf{u}\mathbf{t}\mathbf{o}}$	0.00	0.00	0.01	-0.15
	$\:{\mathbf{h}}_{\mathbf{t}\mathbf{o}\mathbf{t}\mathbf{a}\mathbf{l}}^{2}$	**0.03**	0.02	0.05	0.23
**TURB**	$\:{\mathbf{h}}_{\mathbf{c}\mathbf{h}\mathbf{r}\mathbf{x}}^{2}$	**0.03**	0.01	0.05	0.06
	$\:{{\upsigma\:}}_{{\mathbf{u}}_{\mathbf{c}\mathbf{h}\mathbf{r}\mathbf{x}}}^{2}$	0.02	0.01	0.04	0.07
	$\:{\mathbf{h}}_{\mathbf{a}\mathbf{u}\mathbf{t}\mathbf{o}}^{2}$	**0.04**	0.01	0.09	0.66
	$\:{{{\upsigma\:}}_{\mathbf{u}}^{2}}_{\mathbf{a}\mathbf{u}\mathbf{t}\mathbf{o}}$	0.03	0.01	0.07	0.65
	$\:{\mathbf{h}}_{\mathbf{t}\mathbf{o}\mathbf{t}\mathbf{a}\mathbf{l}}^{2}$	**0.07**	0.03	0.10	0.43
**SC**	$\:{\mathbf{h}}_{\mathbf{c}\mathbf{h}\mathbf{r}\mathbf{x}}^{2}$	**0.07**	0.03	0.11	0.48
	$\:{{\upsigma\:}}_{{\mathbf{u}}_{\mathbf{c}\mathbf{h}\mathbf{r}\mathbf{x}}}^{2}$	0.45	0.21	0.69	0.49
	$\:{\mathbf{h}}_{\mathbf{a}\mathbf{u}\mathbf{t}\mathbf{o}}^{2}$	**0.68**	0.61	0.75	0.17
	$\:{{{\upsigma\:}}_{\mathbf{u}}^{2}}_{\mathbf{a}\mathbf{u}\mathbf{t}\mathbf{o}}$	4.30	3.88	4.71	0.19
	$\:{\mathbf{h}}_{\mathbf{t}\mathbf{o}\mathbf{t}\mathbf{a}\mathbf{l}}^{2}$	**0.75**	0.68	0.81	0.49
**RTL**	$\:{\mathbf{h}}_{\mathbf{c}\mathbf{h}\mathbf{r}\mathbf{x}}^{2}$	**0.05**	0.01	0.10	0.67
	$\:{{\upsigma\:}}_{{\mathbf{u}}_{\mathbf{c}\mathbf{h}\mathbf{r}\mathbf{x}}}^{2}$	0.05	0.01	0.09	0.68
	$\:{\mathbf{h}}_{\mathbf{a}\mathbf{u}\mathbf{t}\mathbf{o}}^{2}$	**0.27**	0.21	0.34	0.33
	$\:{{{\upsigma\:}}_{\mathbf{u}}^{2}}_{\mathbf{a}\mathbf{u}\mathbf{t}\mathbf{o}}$	0.26	0.19	0.32	0.30
	$\:{\mathbf{h}}_{\mathbf{t}\mathbf{o}\mathbf{t}\mathbf{a}\mathbf{l}}^{2}$	**0.32**	0.24	0.34	0.33
**LTL**	$\:{\mathbf{h}}_{\mathbf{c}\mathbf{h}\mathbf{r}\mathbf{x}}^{2}$	**0.05**	0.01	0.12	0.75
	$\:{{\upsigma\:}}_{{\mathbf{u}}_{\mathbf{c}\mathbf{h}\mathbf{r}\mathbf{x}}}^{2}$	0.05	0.02	0.12	0.75
	$\:{\mathbf{h}}_{\mathbf{a}\mathbf{u}\mathbf{t}\mathbf{o}}^{2}$	**0.28**	0.21	0.36	0.39
	$\:{{{\upsigma\:}}_{\mathbf{u}}^{2}}_{\mathbf{a}\mathbf{u}\mathbf{t}\mathbf{o}}$	0.28	0.21	0.36	0.35
	$\:{\mathbf{h}}_{\mathbf{t}\mathbf{o}\mathbf{t}\mathbf{a}\mathbf{l}}^{2}$	**0.34**	0.22	0.36	0.35
**RTW**	$\:{\mathbf{h}}_{\mathbf{c}\mathbf{h}\mathbf{r}\mathbf{x}}^{2}$	**0.04**	0.01	0.07	0.32
	$\:{{\upsigma\:}}_{{\mathbf{u}}_{\mathbf{c}\mathbf{h}\mathbf{r}\mathbf{x}}}^{2}$	0.01	0.00	0.02	0.33
	$\:{\mathbf{h}}_{\mathbf{a}\mathbf{u}\mathbf{t}\mathbf{o}}^{2}$	**0.32**	0.26	0.38	0.07
	$\:{{{\upsigma\:}}_{\mathbf{u}}^{2}}_{\mathbf{a}\mathbf{u}\mathbf{t}\mathbf{o}}$	0.09	0.07	0.11	0.04
	$\:{\mathbf{h}}_{\mathbf{t}\mathbf{o}\mathbf{t}\mathbf{a}\mathbf{l}}^{2}$	**0.36**	0.27	0.39	0.05
**LTW**	$\:{\mathbf{h}}_{\mathbf{c}\mathbf{h}\mathbf{r}\mathbf{x}}^{2}$	**0.04**	0.01	0.07	0.51
	$\:{{\upsigma\:}}_{{\mathbf{u}}_{\mathbf{c}\mathbf{h}\mathbf{r}\mathbf{x}}}^{2}$	0.01	0.00	0.02	0.51
	$\:{\mathbf{h}}_{\mathbf{a}\mathbf{u}\mathbf{t}\mathbf{o}}^{2}$	**0.32**	0.25	0.39	0.20
	$\:{{{\upsigma\:}}_{\mathbf{u}}^{2}}_{\mathbf{a}\mathbf{u}\mathbf{t}\mathbf{o}}$	0.09	0.07	0.11	0.17
	$\:{\mathbf{h}}_{\mathbf{t}\mathbf{o}\mathbf{t}\mathbf{a}\mathbf{l}}^{2}$	**0.36**	0.25	0.39	0.04
**TV**	$\:{\mathbf{h}}_{\mathbf{c}\mathbf{h}\mathbf{r}\mathbf{x}}^{2}$	**0.06**	0.03	0.09	0.00
	$\:{{\upsigma\:}}_{{\mathbf{u}}_{\mathbf{c}\mathbf{h}\mathbf{r}\mathbf{x}}}^{2}$	0.01	0.00	0.01	0.00
	$\:{\mathbf{h}}_{\mathbf{a}\mathbf{u}\mathbf{t}\mathbf{o}}^{2}$	**0.34**	0.27	0.40	-0.02
	$\:{{{\upsigma\:}}_{\mathbf{u}}^{2}}_{\mathbf{a}\mathbf{u}\mathbf{t}\mathbf{o}}$	0.04	0.03	0.00	-0.02
	$\:{\mathbf{h}}_{\mathbf{t}\mathbf{o}\mathbf{t}\mathbf{a}\mathbf{l}}^{2}$	**0.39**	0.27	0.41	-0.03
**TF**	$\:{\mathbf{h}}_{\mathbf{c}\mathbf{h}\mathbf{r}\mathbf{x}}^{2}$	**0.14**	0.09	0.22	0.33
	$\:{{\upsigma\:}}_{{\mathbf{u}}_{\mathbf{c}\mathbf{h}\mathbf{r}\mathbf{x}}}^{2}$	0.00	0.01	0.00	0.45
	$\:{\mathbf{h}}_{\mathbf{a}\mathbf{u}\mathbf{t}\mathbf{o}}^{2}$	**0.16**	0.09	0.15	0.35
	$\:{{{\upsigma\:}}_{\mathbf{u}}^{2}}_{\mathbf{a}\mathbf{u}\mathbf{t}\mathbf{o}}$	0.00	0.01	0.00	0.32
	$\:{\mathbf{h}}_{\mathbf{t}\mathbf{o}\mathbf{t}\mathbf{a}\mathbf{l}}^{2}$	**0.30**	0.15	0.21	0.35
**VESIC_L**	$\:{\mathbf{h}}_{\mathbf{c}\mathbf{h}\mathbf{r}\mathbf{x}}^{2}$	**0.03**	0.01	0.08	0.56
	$\:{{\upsigma\:}}_{{\mathbf{u}}_{\mathbf{c}\mathbf{h}\mathbf{r}\mathbf{x}}}^{2}$	0.07	0.03	0.17	0.56
	$\:{\mathbf{h}}_{\mathbf{a}\mathbf{u}\mathbf{t}\mathbf{o}}^{2}$	**0.15**	0.11	0.20	0.18
	$\:{{{\upsigma\:}}_{\mathbf{u}}^{2}}_{\mathbf{a}\mathbf{u}\mathbf{t}\mathbf{o}}$	0.33	0.23	0.44	0.16
	$\:{\mathbf{h}}_{\mathbf{t}\mathbf{o}\mathbf{t}\mathbf{a}\mathbf{l}}^{2}$	**0.19**	0.11	0.18	0.19
**VESIC_W**	$\:{\mathbf{h}}_{\mathbf{c}\mathbf{h}\mathbf{r}\mathbf{x}}^{2}$	**0.05**	0.03	0.07	-0.05
	$\:{{\upsigma\:}}_{{\mathbf{u}}_{\mathbf{c}\mathbf{h}\mathbf{r}\mathbf{x}}}^{2}$	0.02	0.01	0.02	-0.05
	$\:{\mathbf{h}}_{\mathbf{a}\mathbf{u}\mathbf{t}\mathbf{o}}^{2}$	**0.14**	0.10	0.19	-0.04
	$\:{{{\upsigma\:}}_{\mathbf{u}}^{2}}_{\mathbf{a}\mathbf{u}\mathbf{t}\mathbf{o}}$	0.04	0.03	0.06	-0.03
	$\:{\mathbf{h}}_{\mathbf{t}\mathbf{o}\mathbf{t}\mathbf{a}\mathbf{l}}^{2}$	**0.19**	0.13	0.21	-0.02
**MID**	$\:{\mathbf{h}}_{\mathbf{c}\mathbf{h}\mathbf{r}\mathbf{x}}^{2}$	**0.004**	0.001	0.01	0.76
	$\:{{\upsigma\:}}_{{\mathbf{u}}_{\mathbf{c}\mathbf{h}\mathbf{r}\mathbf{x}}}^{2}$	0.06	0.02	0.14	0.76
	$\:{\mathbf{h}}_{\mathbf{a}\mathbf{u}\mathbf{t}\mathbf{o}}^{2}$	**0.02**	0.01	0.04	0.38
	$\:{{{\upsigma\:}}_{\mathbf{u}}^{2}}_{\mathbf{a}\mathbf{u}\mathbf{t}\mathbf{o}}$	0.36	0.12	0.60	0.38
	$\:{\mathbf{h}}_{\mathbf{t}\mathbf{o}\mathbf{t}\mathbf{a}\mathbf{l}}^{2}$	**0.03**	0.01	0.10	0.39
**MAD**	$\:{\mathbf{h}}_{\mathbf{c}\mathbf{h}\mathbf{r}\mathbf{x}}^{2}$	**0.03**	0.01	0.06	0.16
	$\:{{\upsigma\:}}_{{\mathbf{u}}_{\mathbf{c}\mathbf{h}\mathbf{r}\mathbf{x}}}^{2}$	3.17	0.73	5.62	0.16
	$\:{\mathbf{h}}_{\mathbf{a}\mathbf{u}\mathbf{t}\mathbf{o}}^{2}$	**0.12**	0.07	0.17	-0.05
	$\:{{{\upsigma\:}}_{\mathbf{u}}^{2}}_{\mathbf{a}\mathbf{u}\mathbf{t}\mathbf{o}}$	12.04	6.80	17.27	-0.05
	$\:{\mathbf{h}}_{\mathbf{t}\mathbf{o}\mathbf{t}\mathbf{a}\mathbf{l}}^{2}$	**0.15**	0.11	0.19	-0.22
**TD**	$\:{\mathbf{h}}_{\mathbf{c}\mathbf{h}\mathbf{r}\mathbf{x}}^{2}$	**0.02**	0.005	0.04	0.84
	$\:{{\upsigma\:}}_{{\mathbf{u}}_{\mathbf{c}\mathbf{h}\mathbf{r}\mathbf{x}}}^{2}$	2.45	0.58	5.49	0.84
	$\:{\mathbf{h}}_{\mathbf{a}\mathbf{u}\mathbf{t}\mathbf{o}}^{2}$	**0.10**	0.04	0.15	0.41
	$\:{{{\upsigma\:}}_{\mathbf{u}}^{2}}_{\mathbf{a}\mathbf{u}\mathbf{t}\mathbf{o}}$	12.22	5.36	19.09	0.41
	$\:{\mathbf{h}}_{\mathbf{t}\mathbf{o}\mathbf{t}\mathbf{a}\mathbf{l}}^{2}$	**0.12**	0.08	0.32	0.19
**BULL_FIT**	$\:{\mathbf{h}}_{\mathbf{c}\mathbf{h}\mathbf{r}\mathbf{x}}^{2}$	**0.10**	0.04	0.24	0.75
	$\:{{\upsigma\:}}_{{\mathbf{u}}_{\mathbf{c}\mathbf{h}\mathbf{r}\mathbf{x}}}^{2}$	0.44	0.17	1.05	0.78
	$\:{\mathbf{h}}_{\mathbf{a}\mathbf{u}\mathbf{t}\mathbf{o}}^{2}$	**0.18**	0.03	0.39	0.70
	$\:{{{\upsigma\:}}_{\mathbf{u}}^{2}}_{\mathbf{a}\mathbf{u}\mathbf{t}\mathbf{o}}$	0.83	0.17	1.83	0.78
	$\:{\mathbf{h}}_{\mathbf{t}\mathbf{o}\mathbf{t}\mathbf{a}\mathbf{l}}^{2}$	**0.28**	0.16	0.54	0.10
**ASPEC_SMN**	$\:{\mathbf{h}}_{\mathbf{c}\mathbf{h}\mathbf{r}\mathbf{x}}^{2}$	**0.22**	0.02	0.46	0.37
	$\:{{\upsigma\:}}_{{\mathbf{u}}_{\mathbf{c}\mathbf{h}\mathbf{r}\mathbf{x}}}^{2}$	0.26	0.00	0.51	0.55
	$\:{\mathbf{h}}_{\mathbf{a}\mathbf{u}\mathbf{t}\mathbf{o}}^{2}$	**0.29**	0.01	0.56	0.30
	$\:{{{\upsigma\:}}_{\mathbf{u}}^{2}}_{\mathbf{a}\mathbf{u}\mathbf{t}\mathbf{o}}$	0.34	0.03	0.65	0.43
	$\:{\mathbf{h}}_{\mathbf{t}\mathbf{o}\mathbf{t}\mathbf{a}\mathbf{l}}^{2}$	**0.51**	0.19	0.56	0.01

VOL: Ejaculate volume; VIG: Spermatic vigor; TURB: Spermatic vortex; MOT: Rectilinear progressive sperm motility; SC: Scrotal circumference; LTL: Left testicular length; RTL: Right testicular length; LTW: Left testicular width; RTW: Right testicular width; VESICL: Seminal vesicle length; VESICW: Seminal vesicle width; TV: Testicular volume; TF: Testicular format; MAD: Percentage of sperm cells with major sperm defects; MID: Percentage of sperm cells with minor sperm defects; TD: Percentage of total sperm cells with sperm defects; BULL_FIT: evaluation andrological bull’s fitness; ASPC_ SMN: Evaluation of seminal aspect. The SE for all heritability estimated was < 0.001

For the reproductive performance traits in females, the total heritability (h^2^_total_) estimates ranged from 0.25 for rebreeding of heifers up to 14 months of age (REBA) to 0.48 for probability of pregnancy at 14 months (PP14) and the percentage of the h^2^_total_ explained by autosomes (h^2^_auto_) ranged from 68% (REBA) to 87.5% (PP14). The average percentage of h^2^_total_ explained by the X chromosome SNPs (h^2^_x_) was 16.4% for female reproductive performance traits and ranged from 4% (h^2^_x_: 0.02) for ability to remain productive in the herd at least until four years of age while producing one calf per year (STAY) to 31% (h^2^_x_:0.08) for REBA.

For male fertility traits, the h^2^_total_ estimates were 0.06, 0.05, 0.03, and 0.07 for ejaculate volume (VOL), rectilinear progressive sperm motility (MOT), spermatic vigor (VIG), and vortex (TURB), respectively. Among the traits related to breeding soundness evaluation (BSE), semen quality traits accounted for the second highest proportion of h^2^_x_ relative to h^2^_total_ with an average of 38.75%. The h^2^_x_ estimates of 0.02 (VOL), 0.01 (MOT), 0.02 (VIG), and 0.03 (TURB) represent 38%, 28%, 46%, and 43% of the h^2^_total_.

Regarding semen morphological traits, scrotal circumference (SC) exhibited a h^2^_total_ of 0.75, with 10% explained by h^2^_x_ (0.07). On the other hand, the h^2^_total_ estimate for testicular format (TF) was 0.30, with 48% explained by h^2^_x_ (0.14). In general, h^2^_auto_ estimates ranged from 0.14 seminal vesicle width (VESIC_W) to 0.68 (SC). The h^2^_x_ were generally of low magnitude, except for TF. However, the proportion of h^2^_total_ accounted by the X chromosome markers was 19% on average for the semen morphological traits evaluated in this study. With regards to the estimates of h^2^_auto_ and h^2^_x_, and the proportion of h^2^_x_ in h^2^_total_, the lowest average proportion was observed for semen defects (17%), ranging from 14% for total minor defects (MID) (h^2^_auto_ = 0.02 and h^2^_x_ = 0.004) to 21% for total major defects (MAD) (h^2^_auto_ = 0.12 and h^2^_x_ = 0.03). On the other hand, bull and semen evaluations exhibited the highest proportions of h^2^_x_ relative to h^2^_total_ with an average 39.5%, with 36% for andrological fitness (BULL_FIT) and 43% for seminal aspects (ASPC_SMN), respectively.

### Genome-wide association studies

#### Female fertility and reproductive performance traits

**Fig. 1:**
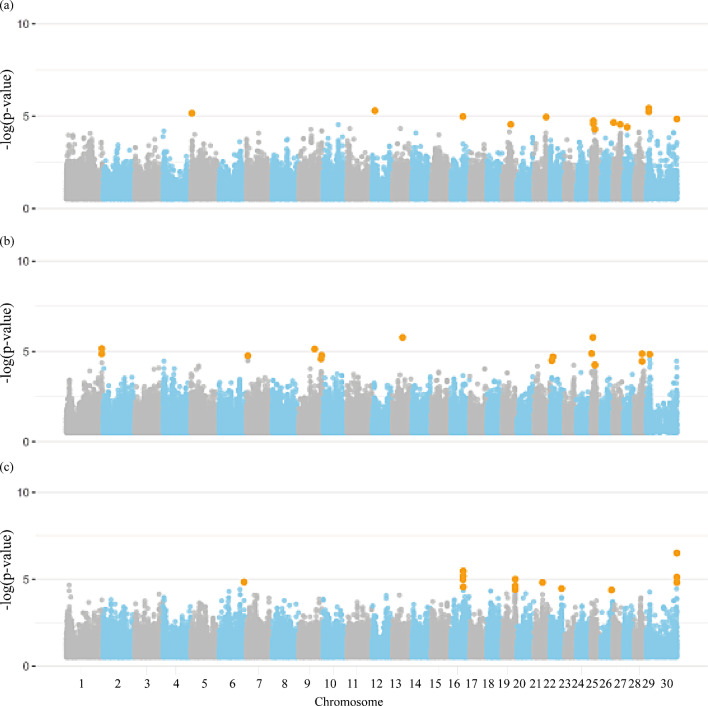
Manhattan plot for the genome-wide association studies (GWAS) of (**a**) re-breeding of heifers up to 14 months, (**b**) rebreeding of females that start reproduction at two years of age, and (**c**) general rebreeding of females throughout their lives in Nellore cattle. Legend: Orange dots indicate the significant markers

**Fig. 2:**
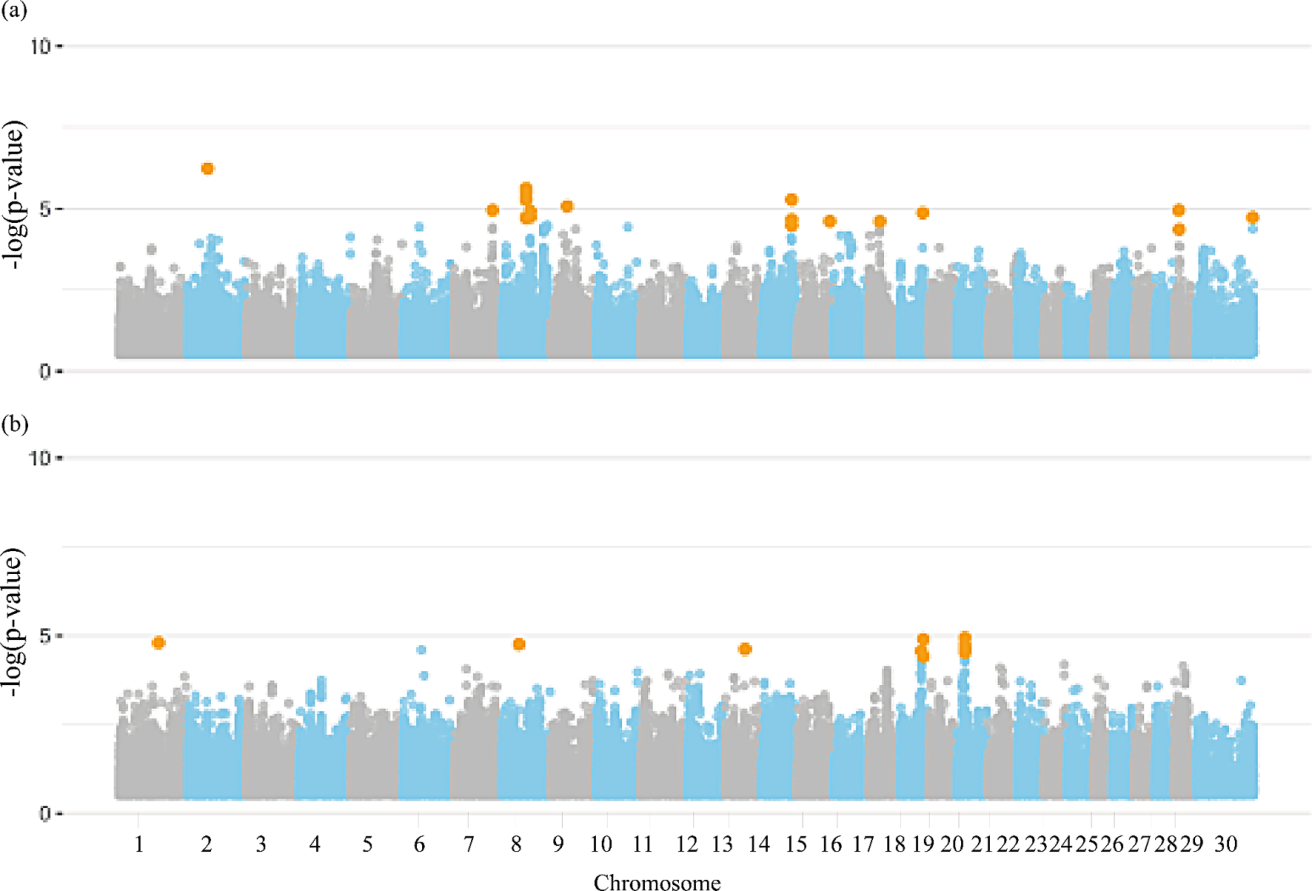
Manhattan plot for the genome-wide association studies (GWAS) of (**a**) probability of pregnancy at 14 months, and (**b**) ability to remain productive in the herd at least until four years, producing one calf per year in Nellore cattle. Legend: Orange dots indicate the significant markers

Figures 1 and 2 present the Manhattan plots for the GWAS of rebreeding and fertility traits in females including all chromosomes in the Nellore population, respectively. A total of 89 significant SNPs were identified for all female traits (Additional file 1: Table S1). Notably, general rebreeding of females throughout their lives (REB), REBA, rebreeding of females that started reproduction at two years old (REBB), PP14, and STAY had 5, 3, 1, 1, and 0 significant SNPs located on the X chromosome, respectively. A total of 683 genes overlapped with genomic regions surrounding the significant SNPs for the female traits, including 328 protein coding genes, 29 long non-coding RNAs, seven microRNAs, four miscellaneous RNA, two processed pseudogenes, two pseudogenes, eight small nucleolar RNAs, and 19 small nuclear RNAs (Additional file 1: Table S2).

Significant genomic regions and candidate genes associated with female reproductive performance traits were selected and are highlighted in Table 3. All other genes found in this study are presented in Additional file 1: Table S2. These candidate genes are associated with sperm/oocyte binding, embryonic development, fertility, calving ease, and regulation of the maturation of fertile oocytes. A significant SNP on BTA25 (16,878,596 bp) harbors the *KNOP1* gene, which was identified for both REBA and REBB. For PP14, three X-chromosome genes (*TMSB4X*, *TLR7*, *PRPS2*) were previously related to environmental adaptation and early reproductive maturity [18, 19].

**Table 3:** Relevant candidate genes and genomic regions, for autosomes and X chromosome, associated for female reproductive performance traits in Nellore cattle

Trait	BTA	Position	Genes
**REBA**	BTA5	4,800,336	*CAPS2*, *GLIPR1L1*, *GLIPR1L2*, *GLIPR1*
	BTA12	12,764,521	*FAM216B*, *EPSTI1*
	BTA16	52,345,056	*TMEM51*
	BTA25	16,878,596	*VPS35L*, *KNOP1*
	BTA25	10,934,871	*BCAR4*
	BTA25	16,878,596	*ARL6IP1*
	BTA27	31,719,740	*KCNU1*
	BTAX	11,581,237	*PRR32*
**REBB**	BTA1	153,941,477	*TBC1D5*
	BTA7	6,884,286	*SMIM7*, *HSH2D*
	BTA9	69,464,242	*ARG1*, *CCN2*
	BTA9	96,459,992	*SOD2*, *ACAT2*, *PNLDC1*, *MAS1*, *IGF2R*, *PLG*
	BTA22	13,606,809	*MYRIP*
	BTA25	17,294,780	*KNOP1*
	BTA25	3,465,680	*SLX4*, *HMOX2*
	BTA25	8,939,538	*GRIN2A*
	BTAX	15,530,039	*STK26*
**REB**	BTA19	56,747,463	*NT5C*, *MRPL58*, *NHERF1*, *GPRC5C*, *TTYH2*
	BTA16	52,345,056	*TMEM51*
	BTA22	49,751,795	*MAPKAPK3*, *CISH*, *HEMK1*, *C22H3orf18*, *CACNA2D2*, *NPRL2*, *CYB561D2*, *ZMYND10*, *RASSF1*, *HYAL1*, *HYAL2*, *HYAL3*, *NAA80*, *IFRD2*, *LSMEM2*, *SEMA3B*, *GNAT1*, *GNAI2*, *SLC38A3*, *SEMA3F*
**PP14**	BTA2	46,972,586	*LYPD6*, *EPC2*
	BTA9	38,349,255	*FYN*
	BTA15	76,977,949	*ARHGAP1*
	BTA18	52,357,363	*PVR*, *BCAM*, *TOMM40*
	BTAX	130,607,937	*TMSB4X*, *TLR7*, *PRPS2*
**STAY**	BTA19	49,142,696	*GMFG*, *PAF1*, *IL-15 L*, *SELENOV*
	BTA19	53,853,761	*PGLYRP1*, *IGFL1*, *ARHGAP35*

REB: All records of rebreeding of females; REBB: Rebreeding of females that entered reproduction at two years old; REBA: Rebreeding of precocity heifers; PP14: Pregnancy probability at 14 months; STAY: Ability to remain productive in the herd

Functional genomic analyses related to these positional genes are presented in Additional file 1: Tables S3 and S4, including 22 KEGG pathways, 88 biological processes, 48 molecular functions, and 25 cellular components. The functional genomic analyses indicated some significant pathways, including “Glutamatergic Synapse” (bta04724) and “Platelet Activation” (bta04611). The significant GO terms “Regulation of Insulin Secretion” (GO:0050796) and “Innate Immune Response” (GO:0045087) associated with female performance reproductive traits.

### Semen quality traits

**Fig. 3:**
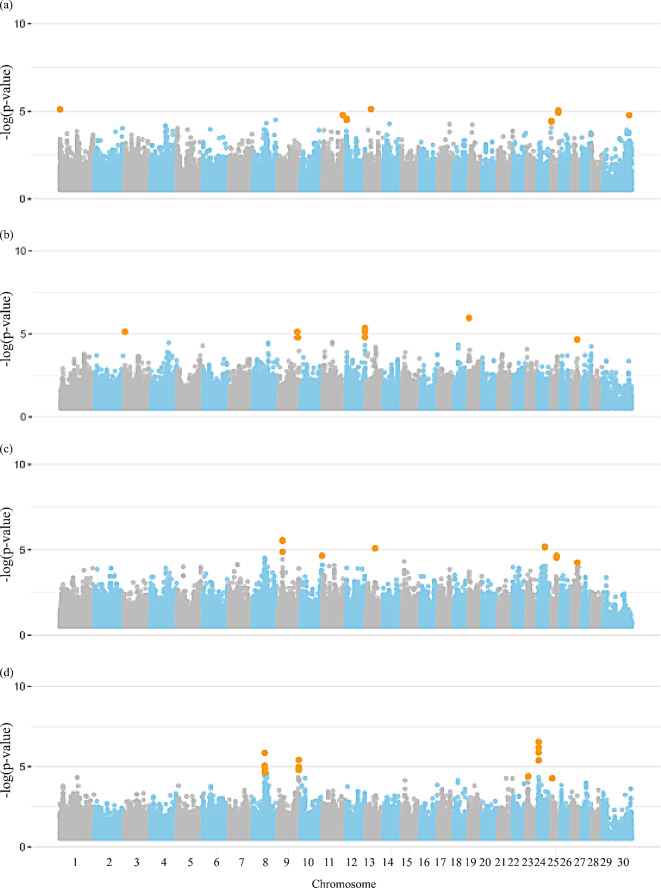
Manhattan plot for the genome-wide association studies (GWAS) of semen quality group of traits, (**a**) ejaculate volume, (**b**) sperm motility, (**c**) spermatic vigor, and (**d**) vortex in Nellore cattle. Legend: Orange dots indicate the significant markers

Figure 3 shows the Manhattan plots for semen quality traits. A total of 252 candidate genes were distributed across all chromosomes. Fifty-five significant SNPs were identified for all semen quality traits group (Additional file 1: Table S5). However, only VOL presented a significant SNP located on the X chromosome (120,877,885 bp). From the total number of candidate genes (*n* = 252), 209 protein coding genes, 17 long non-coding RNAs, six microRNAs, one miscellaneous RNA, three pseudogenes, two ribosomal RNAs, one small nucleolar RNAs, and seven small nuclear RNAs are related to the studied traits. The full characterization of the GO terms is presented in Additional file 1: Table S6.

Several genomic regions were found to be associated with semen quality traits (Table 4 and Additional file 1: Table S7). The identified genes are involved in 47 KEGG pathways, 22 biological processes, 23 molecular functions, and eight cellular components (Additional file 1: Table S6 and Table S8). Some key pathways are “Oocyte Meiosis” (bta04114), “mTOR signaling pathway” (bta04150), and “Progesterone Mediated Oocyte Maturation” (bta04914), which may play a role in maintaining reproductive stability under heat stress conditions [20].

**Table 4:** Relevant candidate genes and genomic regions, for autosomes and X chromosome, associated for semen quality traits in Nellore cattle

Trait	BTA	Position	Genes
**VOL**	BTA1	2,229,077	*CRYZL1*
	BTA13	26,904,228	*MYO3A*
	BTA25	957,796	*SOX8*, *TEKT4*, *IFT140*, *MEIOB*
	BTAX	33,242,005	*TMEM270*
**MOT**	BTA3	1,087,160	*DCAF6*, *ADCY10*
	BTA9	96,459,993	*SOD2*, *TCP1*
	BTA12	85,149,860	*TEX29*
	BTA19	4,267,064	*KIF2B*
**VIG**	BTA9	23,441,779	*PRSS35*, *SNAP91*, *KIAA0408*
	BTA25	24,384,127	*C25H16orf82*, *NSMCE1*
**TURB**	BTA9	98,040,158	*PACRG*
	BTA23	10,377,389	*SLC26A8*, *CPNE5*

VOL: Ejaculate volume; VIG: Spermatic vigor; TURB: Spermatic vortex; MOT: Rectilinear progressive sperm motility

### Semen morphological traits

**Fig. 4:**
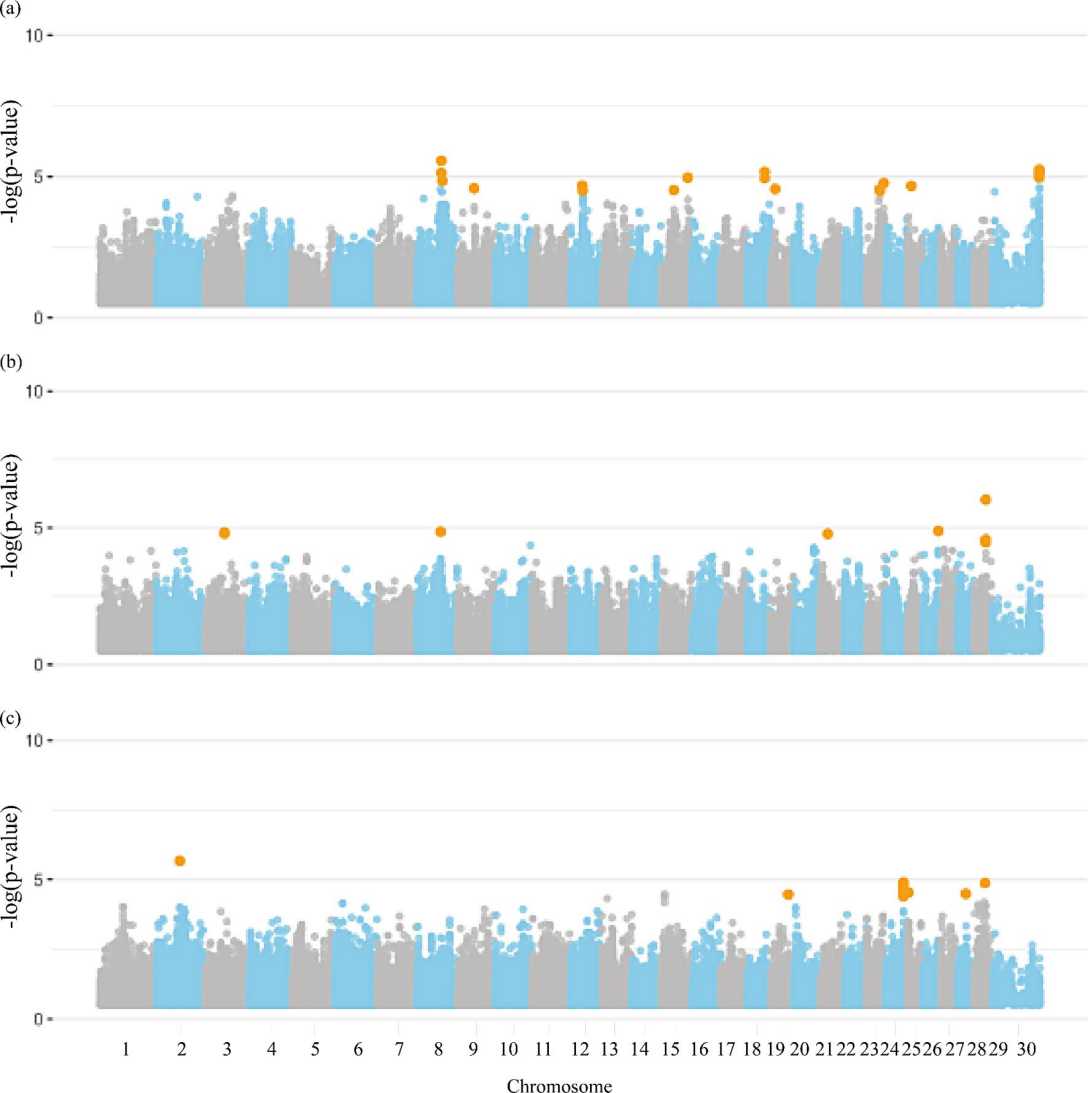
Manhattan plot for the genome-wide association studies (GWAS) of morphological group of traits, (**a**) scrotal circumference, (**b**) testicular volume, and (**c**) testicular shape in Nellore cattle. Legend: Orange dots indicate the significant markers

**Fig. 5:**
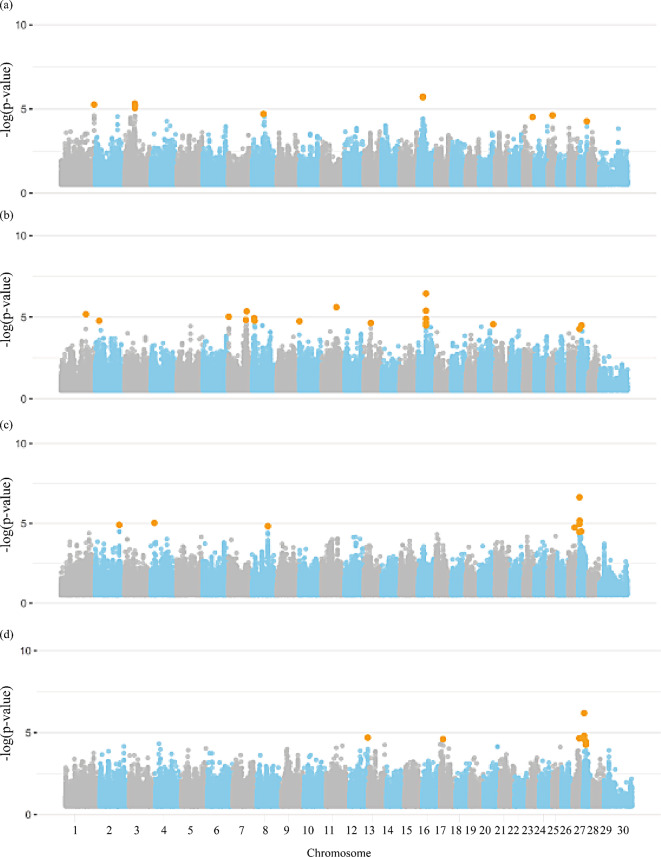
Manhattan plot for the genome-wide association studies (GWAS) of morphological group of traits, (**a**) left and (**b**) right testicular length, left (**c**) and right (**d**) testicular width in Nellore cattle. Legend: Orange dots indicate the significant markers

**Fig. 6:**
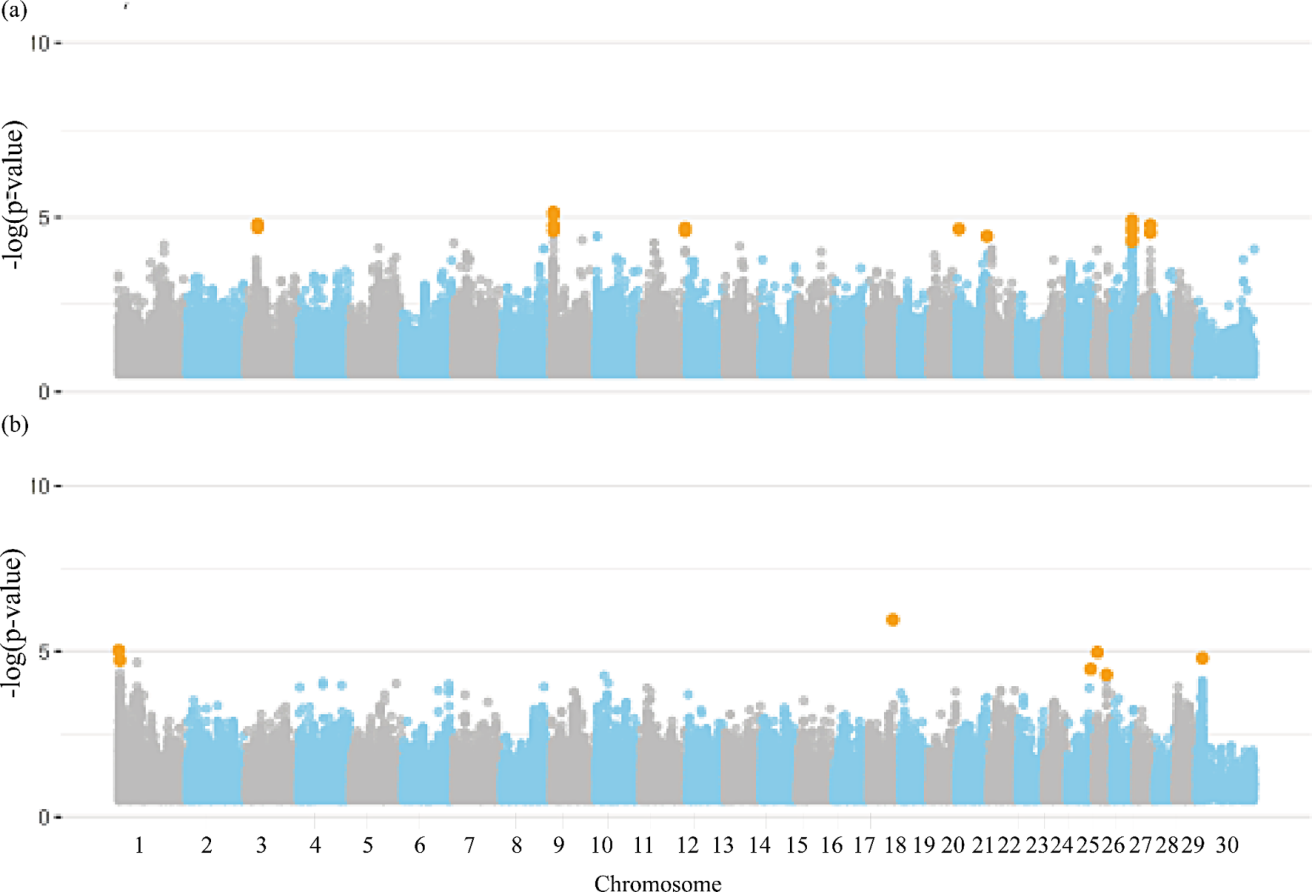
Manhattan plot for the genome-wide association studies (GWAS) of morphological group of traits, (**a**) seminal vesicle length and (**b**) width in Nellore cattle. Legend: Orange dots indicate the significant markers

Figures 4 and 5, and 6 display the Manhattan plots illustrating the significance level of genome-wise SNPs for morphological traits, including the X chromosome. As shown in Additional file 1: Table S9, 124 significant markers were identified for all semen morphological traits. Among these, 11 SNPs were identified on the X chromosome for SC and 1 for VECIC_W. A total of 694 candidate genes, distributed across all chromosomes, were found for all semen morphological traits, including 545 protein-coding genes, 43 long non-coding RNAs, 32 microRNAs, four miscellaneous RNA, eight pseudogenes, 12 ribosomal RNAs, 20 small nucleolar RNAs, and 25 small nuclear RNAs (Additional file 1: Table S10).

Significant associations were identified among all morphological traits and several genes located on different chromosomes (Table 5 and Additional file 1: Table S9). Furthermore, only for SC an association was observed on BTAX with the X-chromosome genes *TMSB4X*, *TLR7*, and *PRPS2*, revealing the role of the X-chromosome genes on the phenotypic variability of SC in Nellore cattle.

**Table 5:** Relevant candidate genes and genomic regions, for autosomes and X chromosome, associated for morphological traits in Nellore cattle

Trait	BTA	Position	Genes
**SC**	BTA12	32,627,792	*GTF3A*
	BTA15	73,648,980	*HSD17B12*, *ALKBH3*
	BTA18	48,837,610	*SIRT2*, *ZFP36*, *SELENOV*
	BTA19	12,208,772	*BCAS3*
	BTA23	37,732,957	*ID4*
	BTAX	130,513,911	*TMSB4X*, *TLR7*, *PRPS2*
**LTL**	BTA8	53,476,203	*GNA14*
	BTA25	21,781,234	*PLK1*, *CHP2*
**LTW**	BTA28	7,602,031	*SLC35F3*, *TARBP1*
**RTL**	BTA1	117,142,191	*GPR87*
	BTA7	3,419,737	*LPAR2*, *PBX4*, *TSSK6*
**RTW**	BTA28	7,602,031	*SLC35F3*
**TV**	BTA3	52,846,895	*LRRC8D*
	BTA8	65,816,013	*TEX10*
	BTA21	23,285,425	*RPS17*, *AP3B2*, *FSD2*
	BTA29	32,722,564	*SPATA19*
**TF**	BTA25	2,831,433	*FLYWCH1*, *HCFC1R1*, *ZNF205*, *ZNF213*, *ZNF200*, *ZNF75A*, *ZNF174*, *ZNF597*, *SLX4*
**VESIC_L**	BTA11	103,282,524	*SPACA9*, *LCN9*, *CAMSAP1*, *LHX3*, *QSOX2*
	BTA20	70,972,905	*TRIP13*
**VESIC_W**	BTAX	13,745,129	*SMARCA1*, *UTP14A*, *BCORL1*

SC: Scrotal circumference; LTL: Left testicular length; RTL: Right testicular length; LTW: Left testicular width; RTW: Right testicular width; VESICL: Seminal vesicle length; VESICW: Seminal vesicle width; TV: Testicular volume; TF: Testicular format

The functional genomic analyses indicated significant pathways and terms associated with the traits, including “Actin Cytoskeleton Regulation” (bta04810), “Thermogenesis” (bta04714), “Leukocyte Trans Endothelial Migration” (bta04670), and “Sperm Flagellum” (GO:0036126). The complete results of the functional genomic analyses for semen morphological traits are presented in Additional file 1: Tables S11 and S12.

### Semen defects

**Fig. 7:**
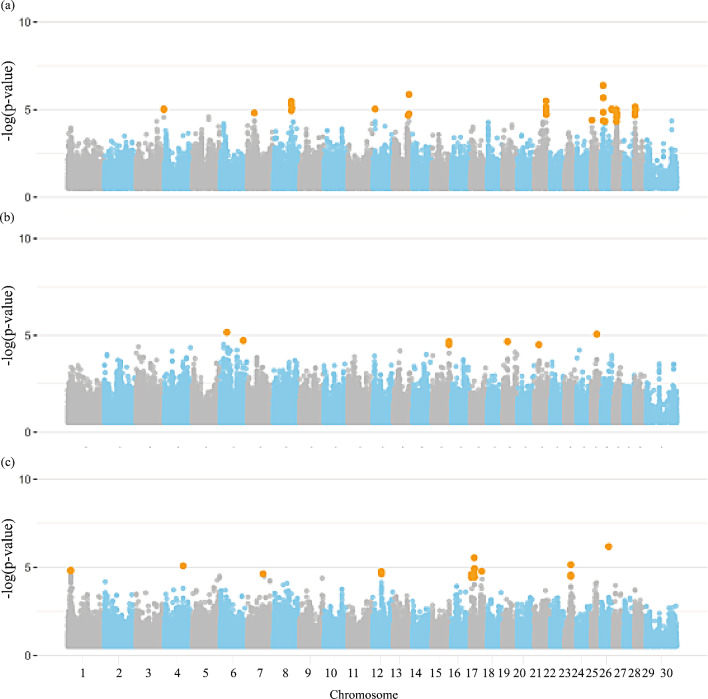
Manhattan plot for the genome-wide association studies (GWAS) of semen defects group of traits, (**a**) total major defects, (**b**) total minor defects, and (**c**) total sperm defects in Nellore cattle. Legend: Orange dots indicate the significant markers

The Manhattan plots illustrating the significance of genome-wise SNPs for semen defect traits are presented in Fig. 7. Seventy-six significant markers were identified for the semen defects group, none of which are located on the X chromosome (Additional file 1: Table S13). Three-hundred-eighty-two candidate genes associated with the traits from this group were distributed across all chromosomes and included 319 protein-coding genes, 21 long non-coding RNAs, 16 microRNAs, four miscellaneous RNAs, four pseudogenes, three ribosomal RNAs, five small nucleolar RNAs, and 10 small nuclear RNAs. The complete list of genes annotated for semen defects is presented in Additional file 1: Table S14.

Some of the genomic regions associated with semen defects are highlighted in Table 6 and Additional file 1: Table S14.

**Table 6:** Relevant candidate genes and genomic regions associated for semen defects traits in Nellore cattle

Trait	BTA	Position	Genes
**MAD**	BTA3	119,362,997	*OR6B2*
	BTA7	30,588,576	*CSNK1G3*
	BTA12	10,793,056	*SUGT1*
	BTA13	65,369,128	*MYL9*, *TGIF2*
	BTA21	53,822,280	*FSCB*
	BTA26	47,083,519	*DOCK1*
	BTA26	10,983,721	*KIF20B*
**MID**	BTA19	22,158,053	*ABR*, *NXN*, *DBIL5*
	BTA25	24,925,889	*NSMCE1*
**TD**	BTA17	5,102,702	*FHDC1*, *ARFIP1*
	BTA23	28,316,603	*TCF19*
	BTA23	28,318,569	*TRIM26*

MAD: Percentage of sperm cells with major sperm defects; MID: Percentage of sperm cells with minor sperm defects; TD: Percentage of total sperm cells with sperm defects

The identified genes are associated with 28 KEGG pathways, 46 biological processes, 14 molecular functions, and 14 cellular components related to semen defects. Furthermore, the functional genomic analyses indicated some significant pathways, including “Antigen Processing and Presentation” (bta04612) and “Glutathione Metabolism” (bta00480). Other relevant pathways and GO terms linked to immune responses were “Autoimmune Thyroid Disease” (bta05320), “Positive Regulation of Immune Response” (GO:0050778), “Defense Response to Virus” (GO:0051607), and “Innate Immune Response” (GO:0045087). The complete list of results from the functional genomic analyses for the semen defects traits studied are presented in Additional file 1: Tables S15 and S16.

### Bulls and semen overall evaluation traits

**Fig. 8:**
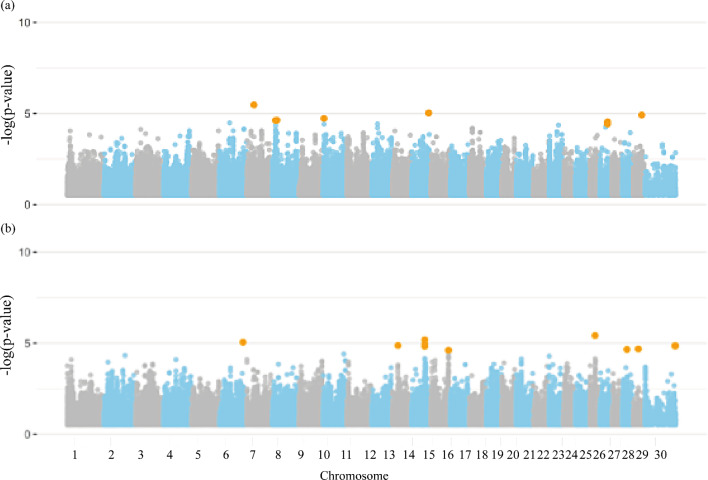
Manhattan plot for the genome-wide association studies (GWAS) of overall bull evaluation group of traits, (**a**) seminal aspects, and (**b**) andrological fitness in Nellore cattle. Legend: Orange dots indicate the significant markers

Nineteen significant SNPs were identified for bulls and overall semen evaluation traits (Additional file 1: Table S17). A significant X chromosome marker (120,877,885 bp) was commonly identified for BULL_FIT and VOL. Sixty-one and 77 candidate genes distributed across all chromosomes, as shown in Fig. 8, were found to be associated with ASPC_SMN and BULL_FIT, respectively. From these, 53 and 61 were classified as protein-coding genes, two and zero long non-coding RNAs, zero and three microRNAs, zero and two miscellaneous RNAs, two and one pseudogenes, two and zero ribosomal RNAs, zero and two small nucleolar RNAs, and one and five small nuclear RNAs, for ASPC_SMN and BULL_FIT, respectively. The complete list of genes annotated for bull and semen overall evaluation traits are presented in Additional file 1: Tables S18 and S19.

For the seminal aspect trait and for the BSE at the end of the andrological period (BULL_FIT), some genomic peaks were found as shown in Table 7 and Additional file 1: Tables S18 to S19. Furthermore, one SNP located on the gene *SMS* in the X chromosome (120,877,885 bp) was also associated with BULL_FIT and VOL.

**Table 7:** Relevant candidate genes and genomic regions, for autosomes and X chromosome, associated for bull and semen evaluation traits in Nellore cattle

Trait	BTA	Position	Genes
**ASPC_SMN**	BTA26	34,182,465	*HABP2*, *TDRD1*
	BTA29	37,919,393	*PAG2*, *PAG5*, *PAG7*, *PAG10*, *PAG12*, *PAG15*, *PAG18*
**BULL_FIT**	BTA6	101,934,743	*PTPN13*
	BTA13	25,169,738	*ARHGAP21*
	BTA15	77,279,691	*NDUFB6*, *PTPRJ*, *PRKCB*
	BTA25	22,338,538	*AQP8*
	BTA28	19,895,096	*REEP3*
	BTAX	120,877,885	*SMS*

BULL_FIT: evaluation of andrological bull’s fitness; ASPC_ SMN: Evaluation of seminal aspect

The genes identified are associated with one and two KEGG pathways, four and two biological processes, five and four molecular functions, and two cellular components, for ASPC_SMN and BULL_FIT, respectively. Furthermore, two GO linked to ASPC, such as “Protein Processing” (GO:0016485), biological process “Zinc Ion Binding” (GO:0008270), and molecular functions associated with semen appearance. For BULL_FIT, a GO term linked to molecular function was identified – “GTPase activator activity” (GO:0005096). The complete functional genomic analyses overview for ASPC_SMN and BULL_FIT is presented in Additional file 1: Tables S20 and S21, respectively.

## Discussion

### Heritability estimates for female reproductive performance and male andrological traits

The X chromosome is commonly removed from genomic analyses primarily due to the unique characteristics of non-PAR (non-pseudo-autosomal region), which differs from the autosomal inheritance mode [21]. In addition, the males (XY) are hemizygous, meaning that they have only one copy of the X chromosome while females have two copies (XX), and dosage compensation mechanisms deactivate one of the two X chromosomes in females [22]. This suggests that both copies of the X chromosome exhibit similar expression, implying a random inactivation of either copy in the females [16]. This study is a follow-up of a previous paper describing genetic parameters for andrological traits and female reproductive performance in Nellore cattle [5]. This current study focuses on the contributions of the X chromosome to the phenotypic variability of male and female fertility and reproduction traits.

In a previous study, the h^2^_total_ estimated for female fertility and precocity traits in Nellore cattle increased by 11.6–18% when including X chromosome SNPs in the analyses with estimates ranging from 0.02 to 0.09 [11]. These results corroborate with those found in this current study with h^2^_total_ estimated for rebreeding and female fertility traits ranging from 0.01 (STAY) to 0.08 (REBA). In dairy cattle, studies focusing on milk production, fertility, health, and conformation traits, reported that the incorporation of X chromosome markers in the analyses led to the capture of higher genetic variation and greater reliability of the genomic estimated breeding values (GEBV) by up to 0.5% and 1%, depending on the model fitted [9, 23]. Therefore, in both dairy cattle and Nellore beef cattle there was a higher capture of genetic variation when including SNPs from the X chromosome on the analysis.

Semen quality traits are heritable with significant additive genetic variation, suggesting that genetic improvement of these traits is feasible [5, 24]. Low heritability estimates captured by the X chromosome markers have been reported for various fertility traits in Brahman and Tropical Composite cattle populations [25]. Conversely, in dairy cattle, the X chromosome influence on milk production, health, and fertility was assessed across different populations and breeds indicating that, on average, ~ 3.5% of the total genetic variance of each trait was explained by the X chromosome markers [26].

The semen morphological traits evaluated in this study showed the highest h^2^_total_ estimates and the lowest proportion of h^2^_x_ in h^2^_total_. However, the inclusion of genomic information from the X chromosome have led to higher h^2^_total_ estimates for semen morphological traits as compared to a previous study in the same population but ignoring the X chromosome information [5]. The inclusion of the X chromosome markers in the genomic analyses of SC resulted in an increase of the h^2^_total_ from 0.02 to 0.09, when compared to models utilizing only autosomal genomic information [11]. The X-linked inactivation status loci could influence the heritability estimates of a trait [27]. Other factors, such as Mendelian sampling and the highly polygenic nature of traits, can also influence heritability estimates [28]. However, it is essential to highlight that these factors are related to genetic variation but do not directly cause it.

Bulls’ fertility is influenced by various environmental factors. Semen quality relies on a sperm free of defects, which is crucial for the bulls’ efficiency in mating seasons [29]. Fortes et al. [25] evaluating semen defects in Brahman bulls, including or not the X chromosome markers in the analyses, reported heritability values ranging from 0.00 ± 0.02 (sperm with abnormal tail) to 0.35 ± 0.07 (normal sperm). In this same study, the heritability estimates for traits related to seminal attributes ranged from 0.09 ± 0.05 to 0.13 ± 0.04 across different Brahman and Tropical Composites cattle populations [25]. This aligns with the findings of this current study, demonstrating the importance of considering X chromosome markers in the analyses, especially when genetically evaluating andrological traits.

Selecting bulls with superior genetic merit for BSE can improve fertility rates, and thus, enhance the overall herd reproductive efficiency [30]. Furthermore, selection of more fertile bulls directly impacts female efficiency and precocity as the genetic correlations among these traits ranged from − 0.46 ± 0.001 (BULL_FIT with REBA) to 0.48 ± 0.001 (VIG with REBA) [5]. Regarding BULL_FIT, a non-negligible proportion of h^2^_total_ was due to the X chromosome (h^2^_x_ = 0.10 ± 0.001). Neglecting the X chromosome information in the analyses of genetic parameters could overestimate autosomal effects. Therefore, including the X chromosome information is crucial for both females and males as also reported in other beef [25] and dairy [26] cattle populations. In summary, most of the male and female reproductive performance and semen traits evaluated in this study are heritable and influenced by genes located on the X chromosome. Furthermore, the use of more robust relationship matrices that include more complete genomic information accounting for relationships based on the X chromosome can provide more accurate estimates [9, 31]. Therefore, information from markers located on the X chromosome should be included in the genetic and genomic evaluations when predicting breeding values for these traits in Nellore cattle.

### Genome-wide association studies

#### Female reproductive performance traits

In general, fertility and reproductive performance in cattle are highly polygenic traits [32–34]. A total of 14, 21, 16, 25, and 13 SNPs were significantly associated with REBA, REBB, REB, PP14, and STAY respectively (Additional file 1 Table S1). One candidate gene overlapped with these genomic regions (Table 5). For REBA, the *Calcyphosine* 2 (*CAPS2*) gene has been described to be involved in obesity regulation during embryonic pre-implantation in sheep [35]. Body condition can influence bovine reproduction through changes in ovarian cell proliferation, cytoplasmic markers of apoptosis, and IGF-I (insulin-like growth factor I), which are key regulators of fecundity in cattle and other species [36].

A family group of genes including *GLIPR1L1*, *GLIPR1L2*, and *GLIPR1*, which were associated with REBA, may facilitate the process of fertilization between sperm and oocyte in mammals [37]. There were two candidate regulatory genes located in BTA12, which were associated with REBA, including *FAM216B* associated with natural pulses of pre-luteolytic prostaglandin F2α in Holstein dairy cattle [38], and *EPSTI1*, which has been reported to be upregulated in bovine endometrium and influence endometrial remodeling and organ development in early pregnancy in crossbred beef cattle [39].

In the BTA25, the candidate gene *Breast Cancer Antiestrogen Resistance 4* (*BCAR4*), associated with REBA in this study, was previously described as a gene differentially expressed in cattle oocytes, whose inhibition is detrimental to embryonic development [40]. The candidate gene *ARL6IP1*, located on the same chromosome, might be linked to the cow’s ability to stay pregnant even during tough conditions such as heat stress [41], which is common to Nellore cows raised in Brazil. Similarly, *KNOP1*, which has been linked with prenatal stress in Brahman heifers. Prenatal stress has the potential to result in long-term phenotypic changes in reproduction performance [42]. This candidate gene overlapped with a SNP located on BTA25 (16,878,596 bp) that was associated with REBB.

The Nellore cow’s precocity and ability to remain productive in the herd in the second calving is associated with REBA. A candidate gene (*VPS35L*) located on BTA25 was found to be associated with age at first calving and calving ease in dairy cattle [43]. In Nellore cattle, *KCNU1* located on BTA27 (31,719,740 bp) was previously associated with calving ease and pregnancy rate of daughters for low environmental conditions [44]. An important X-linked candidate gene (*PRR32*) was observed by combining association results within six breeds in meta-analyses for fertility traits [26].

For REBB, we found a set of nineteen candidate genes with potential roles in various physiological and reproductive processes. *TBC1D5* (BTA1) was previously associated with days from calving to first service in Holstein cows [45]. Candidate genes located on BTA7, such as *SMIM7* and *HSH2D*, have been associated with the development of bovine embryos during the pre-implantation phase in taurine and crossbred cattle [39, 46]. Candidate genes located on BTA9, BTA22, and BTA25 were also previously associated with different aspects of fertility and reproductive processes in cattle. The candidate genes *ARG1*, *CCN2*, *SOD2*, *ACAT2*, *PNLDC1*, *MAS1*, *IGF2R*, and *PLG* (BTA9) have been shown to be involved in different biological functions associated with cattle reproduction. For instance, *ARG1* has been reported to be involved in anti-inflammatory regulation and pregnancy support in crossbred cows [47]; *CCN2* and *IGF2R* influence embryonic development, showing significant expression during pregnancy and at specific stages of fetal development, respectively [48, 49]. The *MYRIP* gene (BTA22) is involved in maternal recognition of pregnancy and implantation [50]. The genes *SLX4*, *HMOX2*, and *GRIN2A* (BTA25) have been associated with fertility traits and sub-indexes in Holstein cattle [45, 51, 52].

The *STK26* gene, located on the X chromosome (15,866,834 − 17,401,780) was identified in a study of runs of homozygosity and showed a significant association with reproductive traits (e.g., age at first calving, early pregnancy in heifers, and recurrence of pregnancy in heifers) in Nellore cattle [53]. The *TMEM51* gene (BTA16) identified for REBA and REBB, with the observed association indicating its potential role in regulating fertility. Banerjee et al. [54], through transcriptomic analyzes of blood cells and plasma metabolites in Angus-Simmental crossbred heifers, identified biological pathways related to non-pregnant groups, providing clues into possible mechanisms underlying these specific fertility traits [54].

Several genes have been associated with REB, such as *NT5C*, *MRPL58*, *NHERF1*, *GPRC5C*, and *TTYH2*, all of which are located on BTA19. These genes influence embryonic development (*NT5C* and *MRPL58*) [55, 56], communication between embryo and mother (*GPRC5C*) [57], fetal inflammatory response (*NHERF1*) [58], and expression patterns during embryonic and fetal development (*TTYH2*) [59], suggesting their contributions to the regulation of reproduction in cattle. Furthermore, 22 candidate genes located on BTA22 have been previously shown to influence the reproduction of heifers from the first to the last insemination [45].

For PP14, the *EPC2* gene located on BTA2 was previously linked with early embryonic development in pigs [60]. On the BTA9 chromosome, the *FYN* gene, associated with thermotolerance, may play a role in the fertilization process and early embryo development in dairy cows [61]. The *ARHGAP1* gene (BTA15) has been shown to be expressed in the endometrium on the 13th day after estrus expression in crossbred heifers, being essential for maintaining pregnancy [62]. Furthermore, the *PVR*, *BCAM*, and *TOMM40* genes (BTA18) have been linked with embryonic development, early pregnancy programming, and fertilizations in different cattle breeds [63–65]. This highlights the X-linked genes *TMSB4X*, *TLR7*, *PRPS2* in association with PP14, indicating the functional diversity and relevance of these genes in regulating reproduction and environmental adaptation. The *TMSB4X* gene has been associated with heat stress and gene expression in cattle oocytes [19, 66]. *TMSB4X* may contribute to the regulation of the response to heat stress during the reproductive period. Furthermore, the *TLR7* gene, which is associated with immune response and disease resistance [18], also has an impact on fertilization and efficient embryo production in both cattle and mice [67].

For STAY, a significant genomic peak overlapping with various candidate genes was found on BTA18. The candidate gene *GMFG* may be involved in the regulation of bacterial infections that lead to infertility in cattle [68]. Meanwhile, the *PAF1* gene, identified in Nellore cows exhibiting a lower age at first calving, demonstrated an upregulation associated with epigenetic modulation within oocytes and cumulus cells [69]. Other genes on BTA18 are involved in various biological functions, such as immune response (*IL-15 L*) [70] and regulation of body fat (*SELENOV*) [71]. *PGLYRP1-IGFL1* was previously associated with higher values of the lifespan and productive lifetime and consequently the production efficiency of dairy cattle [72]. Furthermore, the *ARHGAP35* gene plays a role in embryonic, placental, and fetal development [73]. These findings highlight the relevance of BTA18 in determining the ability of Nellore females to remain productive in the herd by regulating the reproductive performance.

The identified candidate genes are associated with various female reproductive performance traits involved in multiple biological functions, including “Innate Immune Response” (GO:0045087), “Platelet Activation” (bta04611) [74], and “Glutamatergic Synapse” (bta04724).

### Semen quality traits

Eleven, 9, 11, and 24 SNPs were significantly associated with VOL, MOT, VIG, and TURB respectively (Additional file 1: Table S5). The *CRYZL1* gene (identified based on the significant SNP located on BTA1:2,229,077 bp) has been reported to influence sperm quality in Holstein cattle [75]. Given that sperm volume is a component of semen quality, it is reasonable to assume that the *CRYZL1* gene could also influence sperm volume in Nellore bulls. Another gene, *MYO3*, part of the myosin superfamily and located on BTA13, is expressed only in the testicles [76]. Previous research in Holstein bulls has linked this gene to various aspects of sperm development, such as nuclear morphogenesis, acrosomal formation, mitochondrial translocation, and individualization of spermatids during spermiogenesis [77]. These reports indicate that *MYO3* might play similar roles in spermiogenesis in Nellore bulls.

Several candidate genes located on BTA25 (e.g., *SOX8*, *TEKT4*, *IFT140*, *MEIOB*, and *TMEM270*) were previously associated with spermiogenesis and flagellar development in cattle [78], horses [79], and buffaloes [80]. These findings highlight the complexity of both spermatogenesis and ejaculate quality and pinpoint genes with possible role in these biological processes. In terms of genes located on the X chromosome, the *SMS* (Spermine Synthetase) candidate gene has been previously associated with the “Spermine Biosynthesis” pathway related to male fertility [81]. These findings highlight the importance of the metabolic pathways identified, e.g. “Spermine Synthesis”, in regulating spermatogenesis based on genes from the X chromosome and reveals a functional candidate gene for andrological traits. Furthermore, for MOT, genes (located on different chromosomes) associated with sperm motility regulation were identified. The *DCAF6* gene (BTA3) has been reported to influence the transcriptional activity of androgen receptors in Nellore cattle [82]. The *ADCY10* gene (also located on BTA3) encodes soluble adenylate cyclase, fundamental for the regulation of sperm motility in humans [83]. The *SOD2* and *TCP1* genes (BTA9) are associated with the activity of superoxide dismutase and the regulation of spermatogenesis, respectively, positively influencing sperm concentration and motility in beef cattle [6]. On BTA12, the *TEX29* gene expressed in early stages of spermiogenesis has been linked to spermatozoa development and motility, and fertilization capacity in buffalo [80].

For VIG, *SNAP91* (Synaptosome-Associated Protein 91, BTA9) is a key candidate gene that has been previously associated with sexual maturity and spermatogenesis in Wandong cattle (*Bos taurus*) [84]. The SNAP91 protein regulates sperm formation, influencing sperm quality and viability [84]. Furthermore, the *KIAA0408* gene (BTA9) has been reported to affect sperm rich fraction in pigs and fertility rate of artificially inseminated animals [85]. Another relevant gene identified was *C25H16orf82* (BTA25), which has been differentially expressed in semen samples of bulls with good fertility [86]. This finding indicates the influence of *C25H16orf82* on sperm vigor and seminal quality. Furthermore, the *NSMCE1* gene (BTA25) has been previously associated with the initial phases of spermatogenesis, such as spermatogonia proliferation and meiosis in pigs, indicating its potential link with semen quality [87].

For TURB, genes located on BTA9 and BTA23 associated with development, maturity, and sperm functional maintenance were identified. The *PACRG* gene (BTA9) is directly related to the formation and functional stability of sperm flagella [6]. The *SLC26A8* gene (BTA23) plays a crucial role in ionic transport in male germ cells [88]. The candidate *CPNE5* (BTA23) has been shown to influence hormonal regulation and semen quality in goats [89]. Furthermore, for the candidate genes related to semen quality, three KEGG pathways were identified, including “Oocyte Meiosis” (bta:04114), which is involved in sperm protection against premature acrosome reaction and mediate sperm-oocyte interactions [90]; “Progesterone Mediated Oocyte Maturation” (bta:04914), which has been reported to be involved in oocyte maturation and heat stress response in cattle [20]; and the “mTOR Signaling Pathway” (bta:04150), in which genes related to this pathway are involved in the process of establishing pregnancy [91].

### Semen morphological traits

A total of 26, 10, 20, 10, 9, 10, 12, 20, and 7 SNPs were significantly associated with SC, left testicular length (LTL), right testicular length (RTL), left testicular width (LTW), right testicular width (RTW), testicular volume (TV), TF, seminal vesicle length (VESIC_L), and VESIC_W, respectively (Additional file 1: Table S9). One candidate gene overlapped with these genomic regions (Table 7). The *GTF3A* gene located on BTA12 has been previously associated with age at puberty in Nellore bulls, especially age at puberty, being notable in bulls with SC greater than 19 cm [92]. The *ALKBH3* and *HSD17B12* genes (BTA15) were previously associated with fibroblast death and steroid metabolism, highlighting mechanisms involved in the synthesis of testicular steroids in beef cattle [93]. Furthermore, the *SIRT2* gene (BTA18) has a role in testosterone synthesis [94]. The *SELENOV* gene (BTA18) has regulatory functions in reproductive and body metabolism in mice [71]. This gene was also found to be associated with STAY in the present study, showing that it may be involved in both male and female reproductive performance in Nellore cattle. The *BCAS3* gene (BTA19) was associated with the bulls genetic for offspring calving ease and conception rate in heifers [95]. On BTA23, the *ID4* gene commonly expressed in cattle testicular tissues, plays a role in the regulation of spermatogonia stem cells [96].

Candidate genes located on the X chromosome (e.g., *TMSB4X*, *TLR7*, and *PRPS2*) have been previously associated with heat stress response, immune system response, and cattle fertility, respectively [19, 86, 97]. For LTL, the *GNA14* gene (BTA8) has been correlated with protein interaction network for SC and sperm motility in crossbreed cattle [6]. The *PLK1* and *CHP2* genes (BTA25) were identified as significant regulators for high fertility in beef cattle [98], suggesting that bull’s testicular biometry is related to its fertility rate.

For RTL, the *GPR87* gene (BTA1) was previously identified to be associated with the development of body growth functions [99]. On BTA7, genes such as *LPAR2*, *PBX4*, and *TSSK6* were previously related to conformation traits, precocity, and early pregnancy capacity in Nellore cattle, respectively [100–102]. This provides valuable insights into the molecular processes underlying testicular development, testicular morphology, and reproductive capacity in cattle. The *SLC35F3* candidate gene (BTA28), associated with both LTW and RTW, was previously shown to be a promoter of differentially methylated regions associated with age and conception rate in Holstein bulls [95]. This shows the genetic complexity of testicular biometrics influencing reproductive capacity of Nellore bulls. Furthermore, Hodge et al. [103] reported the *TARBP1* gene (BTA28) to be associated with spermatogenesis and male fertility in sheep, which indicates a role in determining testicular biometrics.

The *LRRC8D* gene (BTA3), which is a member of the *LRRC8* gene family and regulates the formation of volume regulation anion channels (VRAC) in cell membranes in sheep [104], was associated with TV. *LRRC8D* plays an important role in regulating cell volume, a vital process for cellular homeostasis and, consequently, reproductive health. The *TEX10* gene (BTA8) is expressed in the bull’s testicle and it has been shown to be associated with the maturation of proteins necessary for sperm migration, and sperm cell interaction [105]. Furthermore, *TEX10* plays a key role in germ cell formation and the acrosome reaction during fertilization, suggesting its importance for the production of fertile sperm [106].

The *RPS17*, *AP3B2*, *FSD2*, and *SPATA19* genes were also associated with TV and sperm functionality in cattle. For instance, *RPS17* (BTA21) was associated with sperm functionality and survival, playing an important role in survival and oxidative stress in bull fertility [97]. *AP3B2* and *FSD2* (BTA21) are related to heat stress response and insemination outcomes in cattle [107]. *SPATA19* (spermatogenesis associated 19, BTA29) is expressed in haploid sperm cells, suggesting its involvement in the mitochondrial organization and/or function of germ cells [96]. The candidate genes *FLYWCH1*, *HCFC1R*, and members of the *ZNFs* family located on BTA25 are associated with TF. These genes are linked with multiple KEGG pathways and gene ontologies related to puberty in young Nellore bulls undergoing andrological evaluation with SC [92]. Zinc finger proteins *(ZNFs)*, abundant in eukaryotes, serve diverse functions as transcription factors, potentially playing specific roles in gene regulation and cell differentiation, pivotal processes in testicular development and function [108, 109]. The *SLX4* gene (BTA25) was previously associated with testicular morphology in male cattle-yak, participating in the formation of double-strand breaks during meiosis and its exclusion is related to defects in spermatogenesis [110]. The expression of this gene may be linked to testicular structure in cattle.

The candidate gene *SPACA9* (BTA11) has been associated with structural deficiencies and morphological abnormalities of sperm in cattle, leading to loss of motility and fertilization function [78]. Likewise, *LCN9* (lipocalin 9) participates (with other genes) in sperm maturation and male fertility [111]. The genes *CAMSAP1* (spectrin-associated proteins regulated by calmodulin) and *QSOX2* are linked to sperm maturation and sperm motility [112], suggesting their relevance in sperm quality and male fertility. Furthermore, the *LHX3* gene (BTA11) was identified in association with VESIC_L. In cattle, it may be associated with sperm function and fertility in bulls [113]. The *TRIP13* gene, located on BTA20, is involved in regulating spermatocytes and the process of sexual maturation in Nellore bulls [92].

Regarding VESIC_W, genes located on the X chromosome such as *SMARCA1*, *UTP14A*, and *BCORL1* overlap with the genomic regions identified. For instance, the *SMARCA1* gene has specific patterns of expression related to biological process in the seminal vesicle of beef cattle [114]. The *UTP14A* gene has been associated with the sex chromosomes inactivation [115] and *BCORL1* is involved in reproductive processes such as spermatogenesis and sperm motility in cattle and rats [116, 117]. The identified genes are related to different KEGG pathways and GO terms, such as the “Regulation of Actin Cytoskeleton” (bta04810), which can influence testicular morphology and cell junction linked to lipid metabolism [118]. “Thermogenesis” (bta04714) has been linked to the regulation of body temperature, which can influence homeostasis and (indirectly) reproductive function in cattle [119]. “Transendothelial Migration of Leukocytes” (bta04670) may be associated with metabolic and protein pathways that impact the immune response, with possible effects on reproductive health described in goats [120]. “Sperm Flagellum” GO term (GO:0036126) indicates the importance of genes in the formation and functioning of the sperm flagellum, which is essential for sperm motility and morphology [120]. These protein markers are intrinsically linked to sperm differentiation and maturation, directly affecting testicular morphology and seminal vesicle biometry.

### Semen defects

A total of 43, 7, and 26 SNPs were significantly associated with MAD, MID, and total sperm defects (TD), respectively (Additional file 1: Table S13). For MAD the *OR6B2* gene located on BTA3 appears to be involved in three modes of sperm activation in humans and mice, and can alter co-expression patterns in sperm with an acrosome reaction [121] where it can play a role in regulating sperm defects. Furthermore, other candidate genes identified have been previously related to immune response in sheep [122] and sperm-related immunological tolerance in pigs [85], such as *SUGT1* (BTA12) and *TGIF2* (BTA13), respectively. In cattle, the *CSNK1G3* gene may be a regulatory gene for some infectious diseases caused by bacterial or viral agents, which can be transmitted through semen and affect fertility [123]. The *MYL9* gene located on BTA13 is linked to non-muscle myosin, which plays roles in cell migration, adhesion, and movement, recognized as a sperm interacting protein (SIP), suggesting its potential involvement in regulating sperm capacity [124].

The *FSCB* gene (calcium binding protein phosphorylated by protein kinase A – BTA25) is related to biogenesis of the fibrous sheath during spermatogenesis; it is expressed in the cortex of the fibrous sheath and has the ability to bind to calcium, influencing sperm flagellar movement [125]. Sigdel et al. [61], in a study with Holstein cattle under heat stress conditions, identified *DOCK1* as a gene related to thermotolerance and possibly involved in the fertilization process. The *KIF20B* gene (BTA26), belonging to the Kinesin family, had increased transcriptional regulation in sperm from bulls with low fertility [126]. *KIF20B* may be involved in intracellular transport processes and may play a critical role in sperm viability and function, indirectly influencing fertility.

For MID, the *ABR* gene (BTA19) associated with bacterial resistance in cattle, encodes a protein that prevents the antimicrobials from reaching the intended target, preventing entry into the cell and competing for space at the binding sites [127]. Its presence among the genes related to MID suggests a possible role in sperm susceptibility to infectious agents. Paiva et al. [128] studying Guzera cattle identified the *NXN* gene to be involved in the response to oxidative stress. Its association with MID suggests a possible role in protecting sperm against oxidative damage.

The *DBIL5* gene (BTA19), an endozepine-like peptide is a specific gene expressed in the testis during the late stages of spermatogenesis [129]. Its association with MID may indicate a delayed sperm maturation, potentially affecting the morphology and viability of these reproductive cells. On the other hand, the *NSMCE1* gene is related to the initial phases of spermatogenesis regulation in pigs, involved in proliferation and meiosis of spermatogonia, in addition to meiotic chromosome segregation [87]. This gene may be crucial for maintaining chromosomal integrity and sperm viability, indirectly influencing semen quality.

For TD, two genes were identified in Holstein-Friesian bulls described in the following study [130], where the *FHDC1* and *ARFIP1* genes, whose expression was identified in sperm coordinate actin and microtubule dynamics, with their protein being enriched in gross motility after freezing. Thus, these genes might influence the quality of post-thawing spermatozoa. The *TCF19* gene was associated with the differentiation of spermatogonia, acting as a regulator of bovine germ cells [131]. The *TRIM26* gene has been reported to modulate host antiviral defense and induce inflammatory immune responses [131].

Pathways related to “Processing and Presentation of Antigen” (bta04612), “Glutathione Metabolism” (bta00480), and “Autoimmune Thyroid Disease” (bta05320) were enriched in this study. bta04612 and bta00480 include proteins present in ejaculated sperm. Additionally, bta00480 has been reported in epididymal fluid and ejaculated sperm, where these proteins function to protect the sperm [132, 133]. bta05320 has been associated with heat stress cellular responses, including immune response [134]. GO terms related to “Positive Regulation of Immune Response” (GO:0050778) were overexpressed in Nellore heifers compared to Angus heifers [135] and “Defense Response to Virus” (GO:0051607) linked to the immune response [136]. These findings indicate that genes associated with semen defects are directly or indirectly related to immune responses.

### Bulls and semen evaluation traits

Eleven and 8 SNPs were significantly associated with BULL_FIT and ASPC_SMN, respectively (Additional file 1: Table S17). The *HABP2* gene (BTA26) was previously linked to seminal quality in cattle. Forouharmehr and Mousavi [137] suggested that the *HABP2* gene may be a candidate gene associated with semen quality, which is a semen attribute trait to optimize the management of cattle breeding herds. Furthermore, the *TDRD1* gene, which is located on the same chromosome, was also identified to have a function associated with regulatory mechanisms of spermatogonia stem cell formation [138]. Another identified genomic region on BTA29 harbors several genes from the *PAG* (Pregnancy-associated glycoprotein) family, including *PAG10*, *PAG2*, *PAG12*, *PAG5*, *PAG18*, *PAG7*, and *PAG15*. Recent studies reported that these genes are highly expressed in ruminants’ placenta and circulating PAG proteins levels are associated with embryonic mortality in cattle [139, 140].

The findings of candidate genes in association with BULL_FIT provide valuable insights into the understanding of molecular and genetic aspects that affect fertility in Nellore bulls. The *PTPN13* gene was shown to influence the sperm growth regulation in cattle [141]. The *ARHGAP21* gene may have an impact on the interval between birth and first service in dairy cattle, regulating important reproductive hormonal functions [142].

Other relevant genes are *NDUFB6* and *AQP8*, which are related to climatic adaptation in cattle. The *NDUFB6* gene have been associated with the respiratory system in animals more adapted to heat stress, which may indicate that this genes may contribute to preventing oxidative stress and cytotoxicity caused by high temperatures [143]. The expression of *AQP8* is stimulated by FSH (Follicle-stimulating hormone) and is highly dependent on hormonal action during puberty [144]. The genotypic variation in the FSH beta subunit gene is associated with sperm abnormalities during spermatogenesis [145].

In short, genetic improvement of bull semen quality and production will contribute to improving the efficiency of artificial insemination (AI) programs and increasing fertility levels. The *PTPRJ* gene can influence the proportion of immune cells, which can lead to higher resistance to diseases in livestock animals [146]. The *PRKCB* gene is potentially associated with sperm function and motility in a Holstein population, which confirms the important role of these genes on semen quality [75]. The *REEP3* gene may be involved in maintaining sperm functions [147]. Furthermore, the *SMS* gene located on the X chromosome was identified for both VOL and BULL_FIT. The *SMS* gene is associated with spermine biosynthesis, playing a crucial role in male fertility [81]. This gene is considered a functional candidate for bovine reproduction, suggesting a X chromosome influence in the regulation of spermatogenesis.

For the functional analysis of genes associated with ASPC_SMN and BULL_FIT, the GO term “Protein Processing” (GO:0016485) was identified in this study. The significant genes identified in the GO term “Zinc Ion Binding” (GO:0008270) was positively regulated in cattle and yaks [6, 117]. This process is potentially involved in reproductive functions, particularly in male fertility, given the crucial role of zinc in sperm physiology [148]. Furthermore, “GTPase Activator Activity” (GO:0005096) may play a role in sperm differentiation [130].

### Implications and next steps

Despite the X chromosome being one of the largest chromosomes in the cattle genome, its information has been ignored in most livestock genomic studies. In this study, we provided a comprehensive investigation of the genetic background of fertility and reproduction traits in Nellore cattle considering genomic markers located in the X chromosome. A large number of genomic regions of small effect were identified, and many candidate genes were highlighted. Future studies should be conducted to further explore these genomic regions in other Nellore populations and other beef cattle breeds. There is still a limited number of genomic markers located on the X chromosome available in the commercial genotyping platforms. Therefore, the use of whole-genome sequence or denser SNP panels could contribute to the identification of additional QTL associated with the studied traits.

## Conclusions

Our findings provide significant evidence of the contributions of the X chromosome on the phenotypic and genetic variability of fertility and reproductive performance traits in male and female Nellore cattle. The total trait heritability proportion ranged from 16.4% (female reproductive performance group of traits) to 39.5% (bull and semen group of traits), indicating that markers located on the X chromosome can capture a substantial proportion of the total additive genetic variance for key fertility and reproduction traits. This underscores the importance of considering X chromosome markers in genomic evaluations of fertility and reproduction traits. Various genomic regions and candidate genes were identified to be associated with the twenty-three traits included in this study. Future studies should further investigate the role of the identified genes on the most relevant fertility and reproduction traits. By elucidating the genetic background of female and male fertility and reproduction traits in Nellore cattle, our findings contribute to the optimization of breeding programs and the sustainability of the beef cattle industry.

## Methods

Institutional Animal Care and Use Committee approval was not needed for this study as all the analyses were performed using pre-existing datasets.

### Phenotypic datasets

The phenotypic records used in this study were collected in seedstock Nellore cattle farms (Agro-Pecuária CFM, São José do Rio Preto, SP, Brazil) located in the Brazilian states of São Paulo and Mato Grosso do Sul. These datasets were managed by the Center for Research in Animal Improvement, Biotechnology, and Transgenics (GMABT) from the University of Sao Paulo (USP, Pirassununga, SP, Brazil). The phenotypic datasets included records from Nellore heifers, cows, and bulls born between 1999 and 2020 and measured at the age of 14 to 48 months. The female traits evaluated in this study were: probability of pregnancy at 14 months (PP14); ability to remain productive in the herd at least until four years, producing one calf per year (STAY); and female re-breeding, including general rebreeding of females throughout their lives (REB), rebreeding of females that started reproduction at two years old (REBB), and re-breeding of heifers up to 14 months of age (REBA).

The male traits evaluated were grouped into four categories: (1) semen quality traits: ejaculate volume (VOL, in mL), vortex (TURB, scale from 0 to 5), rectilinear progressive sperm motility (MOT, in %), and spermatic vigor (VIG, scale from 0 to 5); (2) morphological traits: scrotal circumference (SC, in cm), testicular format (TF, in cm), left and right testicular length (LTL and RTL, in cm), left and right testicular width (LTW and RTW, in cm), testicular volume (TV, in dm^3^), seminal vesicle width (VESICW, in cm) and length (VESICL, in cm); (3) semen defects: total sperm defects (TD, in %), total minor defects (MID, in %), and major defects (MAD, in %); and, (4) overall bull and semen evaluation: andrological fitness (BULL_FIT, scored on a scale from 1 to 4) and seminal aspects (SMN_ASPC, scored on a scale from 1 to 4). All the studied traits are listed in Table 8. The number of observations is equal to the number of phenotypic records after the quality control as each animal had a single phenotypic record. A detailed description of the traits and data editing procedures are presented in Carvalho et al. [5]. The number of animals with phenotypic records and genomic data is presented in Additional File 2: Table S22.

**Table 8:** Summary statistics (after quality control) for male and female fertility and reproduction traits in Nellore cattle

Trait	*N*	Mean (SD)	Mode		Minimum	Maximum	NCG
**Male traits**							
Semen quality							
**VOL (mL)**	15,882	4.07 (2.04)	-		0.50	20.00	617
**VIG (0–5)**	14,361	3.12 (0.54)	3		0	5	586
**TURB (0–5)**	14,877	1.16 (1.10)	0		0	5	608
**MOT (%)**	17,225	70.75(11.40)	-		5	95	606
Morphological traits							
**SC (cm)**	18,435	33.00 (2,64)	-		22.50	48.00	803
**LTL (cm)**	18,693	12.08 (1.27)	-		5.20	18.50	904
**RTL (cm)**	18,680	12.11 (1.25)	-		6.70	19.00	903
**LTW (cm)**	18,677	6.53 (0.67)	-		3.00	9.30	903
**RTW (cm)**	18,662	6.58 (0.67)	-		4.00	9.50	902
**VESIC_L (cm)**	15,054	8.62 (1.93)	-		3.00	16.00	622
**VESIC_W (cm)**	15,038	2.23 (0.61)	-		0.50	10.00	622
**TV (dm** ^**3**^ **)**	15,659	0.25 (0.05)	-		0.06	0.49	631
**TF (1–4)**	18,848	1.85 (0.52)	2		1	4	917
Sperm defects							
**MAD (%)**	14,312	12.75 (10.45)	-		0	131	591
**MID (%)**	13,743	4.67 (4.03)	-		0	67	517
**TD (%)**	14,621	17.33 (11.74)	-		0	136	606
Bulls and semen evaluation							
**BULL_FIT (1–4)**	2,813	1.34 (0.74)	1		1	4	61
**SMN_ASPC (1–4)**	3,839	2.34 (0.94)	2		1	4	118
**Female traits**							
					**%S**		
**REB (1–2)**	65,836	1.52 (0.50)	2	52.10	1	2	191
**REBB (1–2)**	59,675	1.55 (0.50)	2	55.00	1	2	184
**REBA (1–2)**	8,108	1.31 (0.46)	1	31.04	1	2	89
**PP14 (1–2)**	35,057	1.18 (0.39)	1	18.29	1	2	90
**STAY (1–2)**	127,106	1.27 (0.45)	1	28.59	1	2	201

VOL: Ejaculate volume; VIG: Spermatic vigor; TURB: Spermatic vortex; MOT: Rectilinear progressive sperm motility; SC: Scrotal circumference; LTL: Left testicular length; RTL: Right testicular length; LTW: Left testicular width; RTW: Right testicular width; VESIC_COMP; VESICL: Seminal vesicle length; VESICW: Seminal vesicle width; TV: Testicular volume; TF: Testicular format; MAD: Percentage of sperm cells with major sperm defects; MID: Percentage of sperm cells with minor sperm defects; TD: Percentage of total sperm cells with sperm defects; BULL_FIT: evaluation andrological bull’s fitness; SMN_ASPC: Evaluation of seminal aspect; REB: All records of rebreeding of females; REBB: Rebreeding of females that entered reproduction at two years old; REBA: Rebreeding of precocity heifers; PP14: Pregnancy probability at 14 months; STAY: Ability to remain productive in the herd; N: Total of records; SD: Standard deviation; NGCs: Number of contemporary groups; %S: Percentage success rate

Contemporary groups (CGs) for all traits were created considering animals of the same sex, born on the same farm, year, and season, and belonging to the same management group. For all traits, CGs with less than five animals, with progeny from less than two bulls, or formed by animals with unknown pedigree were removed from subsequent analyses. The pedigree file included 660,246 animals (305,484 males and 354,762 females) spanning up to eight equivalent generations.

### Genomic datasets

A total of 11,012 animals, comprising 6,669 males and 4,343 females, were genotyped using the GeneSeek SNP panel (Beadchip Bovine GGP-HDi, Lansing, MI, USA) containing 770,111 single nucleotide polymorphisms (SNPs). Phenotypic performance and pedigree information were available for all genotyped animals. Data from males and females were combined in a unique genotype file. For markers located on the X chromosome, female genotypes were coded as: 0 for animals homozygous for the first allele (AA), 1 for heterozygous animals (AB), and 2 for individuals homozygous for the second allele (BB). For males, hemizygotes for the allele A were assigned a value of 0 and hemizygotes for allele B were coded as 1 [9, 149], which includes both the PAR and non-PAR regions and the SNP information was based on the most recent bovine reference genome assembly (ARS-UCD-1.2).

Genomic quality control (QC) was performed using the PREGSf90 program [150]. Animals and SNPs with a call rate lower than 90% were removed from subsequent analyses. Genotyped animals with more than 1% parent-progeny Mendelian conflicts were also removed. Furthermore, SNPs with minor allele frequency (MAF) lower than 0.05, extreme deviation from Hardy-Weinberg equilibrium (as an indication of genotyping errors) defined by the maximum difference between the observed and expected frequency of heterozygosity higher than 0.15 [151], and duplicated SNPs or those with unknown position were also removed. After QC, 10,574 animals (6,430 males and 4,143 female) with 490,950 SNPs (28,519 SNPs located on the X chromosome and 462,431 SNPs located in autosomal chromosomes) remained for further analyses.

### Statistical analyses

#### Estimation of genetic parameters

Genetic parameters for all traits were estimated via Bayesian inference and the single-step Genomic Best Linear Unbiased Prediction (ssGBLUP) approach [152]. The models used to estimate variance components and genetic parameters for the traits are detailed in Table 9. The effects **a**_(auto)_ and **a**_(X)_ in all the models correspond to the direct additive genetic effects captured by the autosomal and X chromosome markers, respectively, which were used to create the additive genetic relationship matrices for these two random effects; and **e** represents the random residual effects. For REB, REBB, and REBA, the model included the calving rest interval (CRI – in days) of the females, which represents the number of days from calving until the beginning of the second mating: <60 days, > 61 to < 90 days, > 91 to < 120 days, > 121 to < 150 days, and > 151 days.

**Table 9:** Statistical models fitted for the male and female fertility and reproduction traits in Nellore cattle

Model	Traits
**Male traits**	
$\:{\text{y}}_{\text{i}\text{j}\text{k}\text{l}\text{m}\text{n}}=\:{\text{G}\text{C}}_{\text{i}}+{{\upbeta\:}}_{1}{\text{I}\text{D}\text{A}\text{P}}_{\text{j}}+{{\upbeta\:}}_{2}{\text{I}\text{D}\text{A}\text{P}}_{\text{k}}^{2}+{\text{a}}_{\text{l}\left(\text{a}\text{u}\text{t}\text{o}\right)}+{\text{a}}_{\text{m}\left(\text{X}\right)}+{\text{e}}_{\text{i}\text{j}\text{k}\text{l}\text{m}\text{n}}$	VOL; VIG; TURB; MOT; LTL; RTL; LTW; RTW; VESIC_L; VESIC_W; TV; FT; MID; MAD; TD;
	SMN_ASCP; and BULL_FIT
$\:{\text{y}}_{\text{i}\text{j}\text{k}\text{l}\text{m}\text{n}\text{h}}=\:{\text{G}\text{C}}_{\text{i}}+{{\upbeta\:}}_{1}{\text{I}\text{D}\text{A}\text{P}}_{\text{j}}+{{\upbeta\:}}_{2}{\text{I}\text{D}\text{A}\text{P}}_{\text{k}}^{2}+{\text{G}\text{M}\text{A}\text{N}\text{D}}_{\text{l}}+{\text{a}}_{\text{m}\left(\text{a}\text{u}\text{t}\text{o}\right)}+{\text{a}}_{\text{n}\left(\text{X}\right)}+{\text{e}}_{\text{i}\text{j}\text{k}\text{l}\text{m}\text{n}\text{h}}$	SC
**Female traits**	
$\:{\text{y}}_{\text{i}\text{j}\text{k}\text{l}\text{m}}=\:{\text{C}\text{G}}_{\text{i}}+{\text{C}\text{I}\text{R}}_{\text{j}}+{\text{a}}_{\text{k}\:\left(\text{a}\text{u}\text{t}\text{o}\right)}+{\text{a}}_{\text{l}\left(\text{X}\right)}+{\text{e}}_{\text{i}\text{j}\text{k}\text{l}\text{m}}$	REB; REBA; REBB
$\:{\text{y}}_{\text{i}\text{j}\text{k}\text{l}\text{m}\text{l}}=\:{\text{C}\text{G}}_{\text{i}}+{{\upbeta\:}}_{1}{\text{D}\text{T}\text{J}\text{N}}_{\text{j}}+{\text{G}\text{M}\text{A}\text{N}\text{D}}_{\text{k}}+{\text{a}}_{\text{l}\left(\text{a}\text{u}\text{t}\text{o}\right)}+{\text{a}}_{\text{m}\left(\text{X}\right)}+{\text{e}}_{\text{i}\text{j}\text{k}\text{l}\text{m}\text{l}}$	PP14
$\:{\text{y}}_{\text{i}\text{j}\text{k}\text{l}}=\:{\text{C}\text{G}}_{\text{i}}+{\text{a}}_{\text{j}\left(\text{a}\text{u}\text{t}\text{o}\right)}+{\text{a}}_{\text{k}\:\left(\text{X}\right)}+{\text{e}}_{\text{i}\text{j}\text{k}\text{l}}$	STAY

VOL: Ejaculate volume; VIG: Spermatic vigor; TURB: Spermatic vortex; MOT: Rectilinear progressive sperm motility; SC: Scrotal circumference; LTL: Left testicular length; RTL: Right testicular length; LTW: Left testicular width; RTW: Right testicular width; VESIC_COMP; VESICL: Seminal vesicle length; VESICW: Seminal vesicle width; TV: Testicular volume; TF: Testicular format; MAD: Percentage of sperm cells with major sperm defects; MID: Percentage of sperm cells with minor sperm defects; TD: Percentage of total sperm cells with sperm defects; BULL_FIT: evaluation andrological bull’s fitness; SMN_ASPC: Evaluation of seminal aspect; REB: All records of rebreeding of females; REBB: Rebreeding of females that entered reproduction at two years old; REBA: Rebreeding of precocity heifers; PP14: Pregnancy probability at 14 months; STAY: Ability to remain productive in the herd; $\:{\text{y}}_{\text{j}\text{k}\text{l}\text{m}\text{n}}$: are jklmn^th^ vector of phenotypic informations; GC_i_: are i^th^ contemporary groups; IDAP_j_: are j^th^ age measured at 15 months as a covariate; IDAP^2^_k_: are k^th^ quadratic effect of age; GMAND_lk_: are lk^th^ weaning management group as an uncorrelated random effect; CIR_j_: are j^th^ fixed effect of the calving rest intervals; DTJN_j_: are j^th^ fixed effect of the calf birth date; $\:{\text{a}}_{\text{j}\text{k}\text{l}\text{m}}$_(auto)_: are jklm^th^ random animal effects for locus on autosome; $\:{\text{a}}_{\text{j}\text{k}\text{l}\text{m}}$_(X)_: are jklm^th^ random animal effects for locus on X chromosome; $\:{\text{e}}_{\text{j}\text{k}\text{l}\text{m}\text{n}}$: are jklmn^th^ residual random terms

For the random additive genetic effect using the autosomal markers in the ssGBLUP, the inverse of the pedigree relationship matrix (**A**^− 1^) was replaced by the **H**^− 1^ matrix [152], which was calculated as:

$$\:{\mathbf{H}}^{-1}={\mathbf{A}}^{-1}+\left[\begin{array}{cc}0&\:0\\\:0&\:{\mathbf{G}}_{\text{a}\text{u}\text{t}\text{o}}^{-1}-{\mathbf{A}}_{22}^{-1}\end{array}\right]$$

where **H**^− 1^ for autosomes is the inverse of the hybrid relationship matrix (pedigree and genomic information); **A**_22_ is the numerator relationship matrix for the genotyped animals, and **G**_auto_ is the genomic relationship matrix based only on autosomal markers calculated as [153]:

$$\:{\mathbf{G}}_{\text{a}\text{u}\text{t}\text{o}}=\frac{\mathbf{Z}{\mathbf{Z}}^{\mathbf{{\prime\:}}}}{2\sum\:{\text{p}}_{\text{i}}(1-{\text{p}}_{\text{i}})}$$

where **Z** is the gene content matrix containing adjustments for allele frequencies. These factors are fitted to ensure that the mean diagonal of **G**_auto_ is similar to **A**_22_ [154]. For the random effect of genomic relationships based on the X chromosome SNPs ($\:{\mathbf{G}}_{\text{X}}$), the modified pedigree-based relationship matrix **S**, which makes an additional adjustment, considering the differential genetic founders contribution and the differences between the sexes in the transmission of the X chromosome, was calculated as proposed by Fernando and Grossman [149]. A partition based on whether the animals were genotyped or not was considered in the creation of the **S** matrix, which had the following structure:

$$\:\mathbf{S}=\:\left[\genfrac{}{}{0pt}{}{{\mathbf{S}}_{11}}{{\mathbf{S}}_{21}}\genfrac{}{}{0pt}{}{{\mathbf{S}}_{12}}{{\mathbf{S}}_{22}}\right]$$

where ungenotyped (subscript 1) and genotyped (subscript 2) animals were specified to be similar to the pedigree-based relationship matrix. After this, the genomic relationship matrix for the X chromosome markers ($\:{\mathbf{G}}_{\text{X}}$) was created following the method proposed by Druet and Legarra [12]. The gene content for the X chromosome ($\:{\mathbf{M}}_{\text{X}}$) was centered. Specifically, the $\:{\mathbf{M}}_{\text{X}}$ was centered differently for males and females to create the **Z**_X_ matrix: $\:{\mathbf{Z}}_{\text{X}}={\mathbf{M}}_{\text{X}}-1{\mathbf{p}}^{\mathbf{{\prime\:}}}$ for males and$\:\:{\mathbf{Z}}_{\text{X}}={\mathbf{M}}_{\text{X}}-2{\mathbf{p}}^{\mathbf{{\prime\:}}}$ for females, where $\:\mathbf{p}$ are allele frequencies for males and females. This approach enables the construction of the combined relationship matrix for the X chromosome ($\:{\mathbf{H}}_{\text{X}}$), and its inverse was computed directly as Druet and Legarra [12]:

$$\:{\mathbf{H}}_{\text{X}}^{-1}={\mathbf{S}}^{-1}+\left[\begin{array}{cc}0&\:0\\\:0&\:{\mathbf{G}}_{\text{X}}^{-1}-{\mathbf{S}}_{22}^{-1}\end{array}\right]$$

where, $\:{\mathbf{H}}_{\text{X}}^{-1}$ is the inverse of the relationship matrix **H**_X_, $\:{\mathbf{S}}^{-1}\:$is the inverse of **S**, and $\:{\mathbf{G}}_{\text{X}}^{-1}$ is the inverse of $\:{\mathbf{G}}_{\text{X}}$. The alpha (0.90) and beta (0.10) scale parameters were used to construct the matrices for autosomes and X chromosome for all traits. A single Markov Chain via Monte Carlo method (MCMC) was generated with 1,000,000 samples, and the first 250,000 samples were discarded as burn-in. The remaining samples were saved in intervals of 100 samples. Consequently, the inferences were made based on 7,500 samples from the posterior distribution of the parameters. The Gibbs sampling method was applied using the GIBBS1F90 + program [155] for both continuous and categorical traits, in which threshold models [156] were used for the latter. The “boa” R package [157] was used to evaluate the models’ convergence based on the Geweke test (Z-scores between − 1.96 and 1.96 indicated convergence) [158] and visual inspection.

The heritability explained by autosomal markers (h^2^_auto_), X chromosome markers (h^2^_x_), and total heritability (h^2^_total_), for male and female fertility and reproductive performance traits were calculated as:

$$\:{\widehat{\text{h}}}_{\text{a}\text{u}\text{t}\text{o}}^{2}=\frac{{\widehat{{\upsigma\:}}}_{\text{u}\left(\text{a}\text{u}\text{t}\text{o}\right)}^{2}}{{\widehat{{\upsigma\:}}}_{\text{u}\left(\text{a}\text{u}\text{t}\text{o}\right)}^{2}+{\widehat{{\upsigma\:}}}_{\text{u}\left(\text{x}\right)}^{2}+{\widehat{{\upsigma\:}}}_{\text{e}}^{2}}$$

$$\:{\widehat{\text{h}}}_{\text{x}}^{2}=\frac{{\widehat{{\upsigma\:}}}_{\text{u}\left(\text{x}\right)}^{2}}{{\widehat{{\upsigma\:}}}_{\text{u}\left(\text{a}\text{u}\text{t}\text{o}\right)}^{2}+{\widehat{{\upsigma\:}}}_{\text{u}\left(\text{x}\right)}^{2}+{\widehat{{\upsigma\:}}}_{\text{e}}^{2}}$$

$$\:{\widehat{\text{h}}}_{\text{t}\text{o}\text{t}\text{a}\text{l}}^{2}=\frac{({\widehat{{\upsigma\:}}}_{\text{u}\left(\text{a}\text{u}\text{t}\text{o}\right)}^{2}+{\widehat{{\upsigma\:}}}_{\text{u}\left(\text{x}\right)}^{2})}{{\widehat{{\upsigma\:}}}_{\text{u}\left(\text{a}\text{u}\text{t}\text{o}\right)}^{2}+{\widehat{{\upsigma\:}}}_{\text{u}\left(\text{x}\right)}^{2}+{\widehat{{\upsigma\:}}}_{\text{e}}^{2}}$$

### Genome-wide association studies

A total of 490,950 SNPs for 6,430 males and 4,143 female animals were used for the genome-wide association studies (GWAS). We used de-regressed estimated breeding values (dEBVs) as pseudo-phenotypes for the GWAS. dEBVs were calculated based on phenotypic and pedigree information only and using the method proposed by Garrick et al. [159]. For all animals included in this study, assumptions were made regarding dose compensation for the X chromosome. We conducted mixed linear model-based association analyses (MLMA) [160] using the GCTA software [161]. For the GWAS analyses using the GTCA software, hemizygotes males (X chromosome) for the allele A were coded as 0 and hemizygotes males (X chromosome) for the allele B were coded as 2. The coding of the female genotypes remained the same as previously described. The relationship matrix was constructed using the --make-grm option, which included information from the autosomes and X chromosome. We applied the Bonferroni correction method considering the total number of independent chromosomal segments (M_e_) to account for multiple testing. The significance threshold (*P* < 0.05) considered the effective population size (N_e_) of 80 [162, 163], the average length of a chromosome (L in centimorgans– 1 cM, equivalent to 1 Mb) and the number of chromosomes at the chromosome-wise level [164, 165], following the model: $\:{M}_{e}=\frac{2{N}_{e}L}{\text{l}\text{o}\text{g}\left({N}_{e}L\right)}$, where the threshold was -$\:{\text{log}}_{10}\left(\frac{Pvalue}{{M}_{e}}\right)$, with an average value of 4.6.

### Gene annotation and functional genomic analyses

The GALLO R package [166] was used to annotate the SNPs associated with male and female fertility and reproductive performance traits for autosomal and X chromosome loci. A genomic window of 500 Kb upstream and downstream of each significant SNP was used to determine the genomic regions for candidate gene annotation. The genes overlapping with the significant genomic regions were then used for functional genomic analyses, including metabolic pathways and biological process from the Kyoto Encyclopedia of Genes and Genomes (KEGG) and Gene Ontology (GO) terms, using the DAVID tool [167].

## Electronic supplementary material

Below is the link to the electronic supplementary material.

Supplementary Material 1

Supplementary Material 2

## Data Availability

All the data supporting the results of this article are included within the article and in its supplementary files. The raw data cannot be made publicly available, as it is the property of Brazilian Nellore breeding companies, and this information is commercially sensitive. Access to the raw datasets for research purposes can be made to Dr. Jose Bento Sterman Ferraz (jbferraz@usp.br).
