# Supplementary material for: Genetic parameters and genome-wide association studies including the X chromosome for various reproduction and semen quality traits in Nellore cattle
Source: BMC Genomics. 2025 Jan 10;26:26. doi: 10.1186/s12864-024-11193-2 (PMC11720523; doi:10.1186/s12864-024-11193-2)
Supplement: Supplementary file 2 — Supplementary Material 2 [file 12864_2024_11193_MOESM2_ESM.docx]

**ADDITIONAL FILE 2**

| **Table S22.** Number of animals with phenotypic records and genotypes included in the study. | | |
| --- | --- | --- |
| **Trait** | **Number of animals with phenotypic records** | **Number of genotyped animals with phenotypic records** |
| ASPC_SMN | 3,839 | 138 |
| BULL_FIT | 2,813 | 80 |
| VOL | 15,882 | 490 |
| VIG | 14,361 | 471 |
| TURB | 14,877 | 354 |
| MOT | 17,225 | 478 |
| SC | 18,435 | 636 |
| LTL | 18,693 | 546 |
| RTL | 18,680 | 527 |
| LTW | 18,677 | 528 |
| RTW | 18,662 | 528 |
| VESIC_L | 15,054 | 518 |
| VESIC_W | 15,038 | 518 |
| TV | 15,659 | 521 |
| TF | 18,848 | 530 |
| MAD | 14,312 | 453 |
| MID | 13,743 | 417 |
| TD | 14,621 | 452 |
| REB | 65,836 | 124 |
| REBB | 59,675 | 123 |
| REBA | 8,108 | 123 |
| PP14 | 35,057 | 475 |
| STAY | 127,106 | 375 |
